# Discrimination between thermodynamic models of *cis*-regulation using transcription factor occupancy data

**DOI:** 10.1093/nar/gkt1230

**Published:** 2013-11-27

**Authors:** Robert D. Zeigler, Barak A. Cohen

**Affiliations:** Department of Genetics, Center for Genome Sciences and Systems Biology, Washington University School of Medicine in St. Louis, MO 63108, USA

## Abstract

Many studies have identified binding preferences for transcription factors (TFs), but few have yielded predictive models of how combinations of transcription factor binding sites generate specific levels of gene expression. Synthetic promoters have emerged as powerful tools for generating quantitative data to parameterize models of combinatorial *cis*-regulation. We sought to improve the accuracy of such models by quantifying the occupancy of TFs on synthetic promoters *in vivo* and incorporating these data into statistical thermodynamic models of *cis*-regulation. Using chromatin immunoprecipitation-seq, we measured the occupancy of Gcn4 and Cbf1 in synthetic promoter libraries composed of binding sites for Gcn4, Cbf1, Met31/Met32 and Nrg1. We measured the occupancy of these two TFs and the expression levels of all promoters in two growth conditions. Models parameterized using only expression data predicted expression but failed to identify several interactions between TFs. In contrast, models parameterized with occupancy and expression data predicted expression data, and also revealed Gcn4 self-cooperativity and a negative interaction between Gcn4 and Nrg1. Occupancy data also allowed us to distinguish between competing regulatory mechanisms for the factor Gcn4. Our framework for combining occupancy and expression data produces predictive models that better reflect the mechanisms underlying combinatorial *cis*-regulation of gene expression.

## INTRODUCTION

Regulated gene expression lies at the heart of many biological processes including development (1,2), differentiation (3) and environmental responses (4–6). Often, changes in gene expression occur by one or more transcription factors (TFs) binding to transcription factor binding sites (TFBS) and either enhancing or inhibiting the recruitment of RNA polymerase II to gene promoters (7–10). When multiple TFBS are present in a gene’s promoter, it is difficult to predict the resulting expression of the gene. This is because TFs may function independently of one another (11), or may exhibit one of a number of non-linear interactions, including cooperativity (12), anti-cooperativity (13) or competition (14). An important goal in functional genomics is to produce models that accurately predict patterns of gene expression as a function of changes in the TFBS composition of gene promoters.

Investigators have attempted to learn the binding site specificities of TFs through a variety of methods, including the analysis of promoters of suspected targets (15–18), the analysis of sequences bound *in vivo* by the TF using chromatin immunoprecipitation assays (ChIP-Chip, ChIP-seq) (19–24) and through *in vitro* binding studies (25–27). These studies contribute the important first step of predicting which sequences are likely to be bound by a particular TF.

Recently, some investigators attempted to correlate whole-genome expression profiles and experimentally determined (28–30) or predicted (31,32) occupancy data to the DNA content of regulatory sequences using models based on statistical thermodynamics. In this approach, a gene promoter is modeled as existing in one of a number of states. Each state represents one of all possible configurations of bound and unbound TFs. Each TF in turn is modeled as having either a favorable or an unfavorable interaction with RNA polymerase and the TFBS. By summing over all possible promoter states, it is possible to estimate the fraction of time RNA polymerase spends bound to any given promoter, which is an approximation of the transcription level of the promoter (33). The results of these studies demonstrate that statistical thermodynamic models of transcription are a reasonable framework for producing predictive models of steady-state levels of gene expression that also help explain the mechanisms that underlie *cis*-regulation.

The main difficulty with the thermodynamic approach is parameterizing the models given the available genomic data. Even for a promoter with a relatively small number of TFBS, the number of possible promoter states is immense compared with the number of gene expression observations that can be made, even with high-throughput techniques. Moreover, we usually do not know the identities of all TFs that influence any particular gene promoter. An alternative approach is the use of synthetic promoters composed of defined TFBS. In this approach, promoters with combinations of known TFBS are synthesized and then used to drive the expression of fluorescent reporter genes or sequence barcodes (34–42). The synthetic approach allows investigators to sample large numbers of similar promoters, which provides a simplified system relative to the genome. Although synthetic promoters do not capture all of the intricate features of genomic promoters, they are useful tools for obtaining the statistical power necessary to isolate and quantify the effects of particular promoter features. By focusing on the effects of particular combinations of TFBS, synthetic promoters can be used to parameterize thermodynamic models of *cis*-regulation.

Previous synthetic promoter approaches used only expression data to infer relationships between the TFBS content of gene promoters and the gene expression levels they drive. For example, the model from (36) performs well on the given data, explaining ∼60% of the gene expression variance. However, the degree to which the model accurately describes the underlying biophysical mechanisms responsible for the observations remains an open question.

We sought to extend the synthetic promoter approach by developing a ChIP-based metric of TF occupancy on synthetic promoters. We applied this approach to libraries of binding sites for TFs responsive to the standard 2% glucose condition (43) and conditions in which cells are starved for amino acids (44–46). We used both TF occupancy data and expression data to model the TF–DNA and TF–TF interactions that underlie regulation in this system. Our results demonstrate how occupancy data combined with expression measurements can uncover biophysical processes that underlie gene expression. In particular, interactions that we could not capture with expression data alone were revealed when we combined expression data with occupancy data in a formal biophysical framework.

## MATERIALS AND METHODS

### Construction of strains

Strain BC905 (Mat alpha, his3Δ1 leu2Δ0 lys2Δ::BirA ura3Δ0) was created by integrating BirA into the genome of strain BY4742 (Mat alpha, his3Δ1 leu2Δ0 lysΔ ura3Δ0) at the *lys2* locus via PCORE (47). Briefly, a cassette containing KAN and *URA3* (PCORE) was inserted into the *lys2* locus using primers RZ131 and RZ132 (Supplementary Table S2) and standard transformation protocols (48) with selection on G418. A BirA cassette was created with homology to the *lys2* region by polymerase chain reaction (PCR) amplification using the primers RZ133 and RZ134 (Supplementary Table S2) and plasmid prs313-BirA-NLS (49) as a template. This cassette was used to replace PCORE at the *lys2* locus by transformation with counter-selection on 5-FOA. Insertion was verified by PCR around the upstream and downstream regions of integration (primers RZ147–RZ149, Supplementary Table S2) and by Sanger sequencing.

*CBF1*, *GCN4*, *MET31* and *NRG1* were C-terminally tagged with the myc-C-avi tag by amplifying myc-C-avi with a KAN cassette from plasmid PUG6-myc-C-avi (49) using primer pairs referred to in Supplementary Table S2: RZ129 and RZ130 (*CBF1*), RZ137 and RZ138 (*GCN4*), RZ135 and RZ136 (*MET31*) and RZ127 and RZ128 (*NRG1*) and transforming the resulting PCR product into BC905 using G418 selection to create strains BC906 (BC905 + *CBF1*::*myc-C-Avitag)*, BC907 (BC905 + *GCN4*::*myc-C-Avitag)*, BC908 (BC905 + *MET31*::*myc-C-Avitag)* and BC909 (BC905 + *NRG1*::*myc-C-Avitag)*. Insertion was verified by PCR (Supplementary Table S2, primers RZ92-RZ99, RZ143, RZ144) and by Sanger sequencing. The resulting strains were backcrossed to BY4741, sporulated and offspring-selected, which matched the appropriate genotype (MAT alpha his3Δ1 leu2Δ0 lys2Δ::BirA ura3Δ0 *CBF1*::myc-C-avi KAN). Retention of the tag and BirA was verified by PCR post-mating.

### Media

All strain growth was done in YPD; synthetic complete medium with 2% glucose (SC); synthetic complete medium lacking uracil with 2% glucose (SC-Ura); synthetic complete medium lacking Trp with 2% glucose (SC-Trp); minimal medium + 2% glucose with 300 µM his, 1 mM lys, 2 mM leu, 400 µM Trp (Min); minimal medium + 2% glucose with 300 µM his, 1 mM lys, 2 mM leu, 200 µM Ura (Min + Ura-Trp); or in these same media supplemented with 0.9 µM biotin [YPDB, SCB, SCB-Ura (glucose), MinB, MinB + Ura-Trp].

### Synthetic promoter library creation

Libraries of synthetic promoters were created as described previously (35,36). Briefly, oligos with recognition sites for Cbf1, Gcn4, Met31 and Nrg1 (Supplementary Table S2, RZ84–RZ91) were annealed, then mixed in ratios inversely proportional to the melting temperatures of the annealed products and ligated together. The ligation products were size selected with YM100 Microcon columns and cloned into plasmid pJG102 (36) and maxiprepped. The resulting plasmid was digested to produce a linear product with flanking homology to *TRP1*. The linear product was integrated into the avi-tagged strains following standard large-scale transformation protocols (50). Ten 96-well plates of colonies were picked for each tagged strain, which were subjected to three rounds of dilution purification consisting of growing the strains overnight in SC-URA, then pinning them onto SC-URA agar plates and allowing them to grow for 2 days. The final strains were replica-plated onto SC-Trp to check for misintegration events. Promoter sequences were determined by PCR-amplifying the promoters using double-barcoded primers with restriction sites, followed by a pooled ligation of the PCR products to add Illumina sequencing adapters and then sequencing the final product on the Illumina MiSeq platform. The sequence reads were mapped back to their originating well and plate via the double barcodes with a custom python script. See Supplementary Methods for more details.

### Growth conditions

For expression measurements, strains were grown in glucose and amino acid starvation (AAS) conditions as described previously (36) with the addition of 0.9 µM biotin to all media. For ChIP measurements, strains were grown as for expression in 96-well format overnight. For the glucose condition, 30 µl of overnight culture from each well for a given tagged factor was pooled together, and 20 ml of this pooled culture was added to 980 ml of SCB-Ura (see media) and grown for ∼4.5 h to a final optical density (OD660) of 0.6–1.0. For the AAS condition, growth was carried out as for expression measurements except that after growth to mid-log phase in glucose, 30 µl of each strain for a given tagged factor was pooled together and 20 ml of the pooled culture was spun down briefly (2 min at 1000 G) and the supernatant decanted. The pellet was resuspended in 10 ml of MinB (see media) and added to 990 ml of MinB media. Final OD660 after 6 h of growth was between 0.8 and 1.2.

### YFP expression measurements

Strains were grown as described, and then fixed by adding 4% paraformaldehyde solution (4% formaldehyde, 100 mM sucrose) to a final concentration of 1%. Yellow Fluorescent Protein (YFP) intensities were measured by flow cytometry on a Beckman Coulter Cell Lab Quanta SC. The final expression measurement was the median of at least 10 000 observations of the ratio of raw fluorescence to volume of the cell, as reported as the ‘electronic volume’ by the instrument, normalized to the mean expression of three to four no-insert control promoters on the same plate. The expression for promoters with <80% of counts with fluorescence intensity between 10 and 900 raw fluorescence units was treated as missing data in downstream analyses.

### Biotin-ChIP

Synthetic promoter-containing strains were pooled and fixed with 1% final concentration of formaldehyde, quenched with glycine, washed and the resulting cell pellet was frozen at least overnight at −80C. Pellets were thawed and resuspended in lysis buffer [50 mM HEPES, 150 mM NaCl, 1 mM ethylenediaminetetraacetic acid (EDTA), 1% v/v Triton X-100, 0.1% w/v sodium deoxycholate, 0.1% w/v (sodium dodecyl sulphate) SDS] with protease inhibitor (Roche #11836170001), then bead beaten and sonicated. The supernatant was clarified by centrifugation, then applied to phosphate-buffered saline-washed Dynal M280 streptavidin-coated magnetic beads (Life Technologies, 112-05D) and incubated for 1 h. The supernatant was removed and set aside as input. The beads were washed twice for 5 min per wash in each of lysis buffer, high salt lysis buffer (50 mM HEPES, 0.5M NaCl, 1 mM EDTA, 1% v/v Triton X-100, 0.1% w/v sodium deoxycholate), LiCl wash buffer (500 mM LiCl, 1% NP-40 alternative, 10 mM Tris, pH 8.0, 1 mM EDTA), SDS wash buffer (10 mM Tris, pH 8.0, 1 mM EDTA, 3% SDS) and TE (10 mM Tris, pH 8.0, 1 mM EDTA). The beads were resuspended in 250 µl TE + 0.5% SDS + 10 µl of 20 mg/ml Proteinase-K (NEB P8102S) and distributed into three 250 µl PCR tubes. Then 72.5 µl of input material was combined with 72.5 μl of TE + 1% SDS to which 10 µl of 20 mg/ml proteinase-K was added and distributed into two or three 250 μl PCR tubes per replicate. The tubes were incubated for 4 h at 42°C, 2 h at 72°C and 6 h at 65°C. The material from each replicate was recombined and purified via ChIP cleanup columns (Zymo D5205), eluting in 40 µl of elution buffer. ChIP success was validated by quantitative PCR (qPCR) analysis of known targets for each TF. See Supplemental Methods for a complete description of the ChIP and qPCR protocols.

### Sequencing of ChIP synthetic promoters

ChIPed synthetic promoters were sequenced by adding adapter sequences to synthetic promoters in the input and IP samples via PCR amplification using 23 µl of IP material with 1 µl each of 10 µM primers that were barcoded in the forward read based on sample identity and in the reverse read based on the identity of the tagged TF (Supplementary Table S1) and three different starting concentrations of input material. PCR products between 150- and 600-bp long were gel-purified on a 1.5% TAE agarose gel. Input samples were retained on the basis of similar gel intensities to the corresponding IP sample as an approximate concentration measure. The resulting samples were combined, ethanol precipitated and reconstituted in 30 µl of water. The forward sequencing adapter was added by digestion/ligation exactly as for library sequencing. The final concentration of sequenceable fragments was determined by qPCR using SYBR Green QPCR master mix, primers RZ259 and RZ260 and eight synthetic promoter standards, diluted across five orders of magnitude. The material was sequenced on the Illumina HiSeq 2000 platform using one lane of a paired-end 101-bp run.

### Occupancy of synthetic promoters

The relative occupancy of synthetic promoters was determined by mapping each sequenced read back to its synthetic promoter of origin. First, the read was parsed to determine which binding sites were present. This information was used to map the read back to the originating promoter. The read counts were normalized by the total number of reads that were associated with a given tagged-factor strain, condition and sample type (IP or input). The ratio of normalized IP counts to normalized input counts for a particular promoter was divided by the median normalized IP/input ratio of all promoters lacking a binding site for the ChIPed factor to give the normalized relative occupancy. Scaling to the median background occupancy effectively scales the occupancy values relative to the non-specific binding of the factor. This places all occupancy values from all factors and conditions on the same relative scale, assuming that the non-specific binding distribution is the same for all factors. For demonstrating technical replicate variance, the occupancy was calculated separately for each replicate. For modeling purposes, the replicates were generally combined by summing the promoter coverage across replicates and computing occupancy from the summed values. The exception was Gcn4 in AAS where a single ChIP replicate was used due to substantial depletion of promoters with four or more binding sites in the input of two of the replicates. Promoters with <50 reads in the inputs were excluded from the analysis.

### Thermodynamic model of transcription and TF occupancy

To model gene expression and TF occupancy, we used a thermodynamic model of transcription described previously (28,35,36). In this model, each promoter comprises a set of TFBS, which can exist in one of two states: unbound, or bound by its specific TF. Non-specific binding is not modeled. An implicit binding site is assumed for RNA polymerase, which is also allowed to be bound or unbound to that site. In this model, every promoter exists as collection of states, with each state describing a particular configuration of TFs bound to the DNA. When two TFs are bound to DNA in the same state, they may interact favorably (cooperativity), unfavorably (anti-cooperativity) or not at all (independent binding). These interactions may occur between TFs or between TFs and polymerase. The model treats unbound DNA as a reference state and computes the statistical weight of each possible state that can be produced with *k* TFs*.* The weight of any particular state is the sum of ΔGs of protein–DNA and protein–protein interactions that occur in that state. The statistical weight of a given state divided by the sum of the statistical weights of all possible states is the probability of that state occurring. The occupancy of any particular TF is the sum of the probabilities of all states in which that TF is bound. Likewise, the probability of polymerase binding to a given promoter is the sum of the probabilities of all states in which polymerase is bound. As implemented, the model assumes that the probability of polymerase binding is related to the observed expression by a constant scaling factor. See Supplementary Methods for a complete description of the model.

To fit models to our data, we used a non-linear fitting routine (nlminb, *R* statistical package) to find values for the ΔGs in the model that maximize the correspondence between expression (and/or TF occupancy) predicted by the model and the experimentally measured expression (and/or TF occupancy). This problem is tractable because we assume that the ΔG of any particular protein–DNA or protein–protein interaction is constant across all synthetic promoters. For example, in this work, we were fitting models that contained between 6 and 15 different ΔGs (Supplementary Table S5) using experimental measurements from between 114 and 291 synthetic promoters (Tables 1 and 2). Models were initially fit with only TF–DNA and TF–polymerase interactions: all TF–TF interactions were constrained to 0. In subsequent rounds of fitting, we added each TF–TF interaction to the model, in turn, and then tested for statistically significant differences in the fit relative to a fit without the interaction. Only TF–TF interactions that resulted in significantly better fits were retained in the final models.

**Table 1.** Summary of usable promoters for expression analysis

Tagged TF	Total, glucose	Unique, glucose	Total, AAS	Unique, AAS
Cbf1	529	218	374	125
Gcn4	614	213	396	114
Met31^a^	643	271	475	170
Nrg1^a^	634	271	393	139

Cbf1, Gcn4, Met31 and Nrg1 were tagged with the myc-C-avi tag in a strain harboring the bacterial biotin ligase BirA.

Synthetic promoters containing sites for all four factors were constructed in each strain. Nine hundred sixty colonies were picked for each library, purified, sequenced and then grown in glucose and AAS. The library members were cross-linked, and then run on a Beckman Coulter Cell Lab Quanta SC flow cytometer to measure the fluorescence of the reporter gene in each strain. The numbers shown are the number of strains for which sequence information was determined and for which a reliable fluorescence value was obtained. ^a^Omitted from expression analysis due to lack of ChIP signal from occupancy analysis.

**Table 2.** Summary of usable promoters for occupancy analysis

Tagged TF	Glucose	AAS
Cbf1	290	291
Gcn4	199	229
Met31^a^	0	0
Nrg1^a^	0	0

ChIP was performed on the libraries of synthetic promoters and the promoters specifically sequenced as described in Methods. Promoters with <50 reads in the input replicates were discarded. Met31 and Nrg1 showed no specific enrichment, so all promoters were discarded. The table summarizes the total number of promoters used for analysis for each factor and condition.

^a^No observable ChIP signal.

### Competitive binding model

The competitive binding model functioned exactly as the standard model except that each Gcn4 site had three possible states: unbound, bound by Gcn4 and bound by the unidentified competitor. No direct interaction between Gcn4 and the competitor was modeled. The competitor was assumed to have the same concentration and the same effect on polymerase in both conditions. The Gcn4 effect on polymerase was held constant in both conditions, but its concentration in both conditions was allowed to vary. All other parameters were fit as for the non-competitive model.

### Cross-validation of models

All models were subjected to 5-fold cross-validation. The promoters and associated expression or occupancy values were randomly partitioned into five equally sized sets. In each round of cross-validation, training was performed on four of the five sets of data, and validation was performed on the fifth set of data. Each partition was used once and only once for validation.

## RESULTS

### Promoter libraries with tagged TFs show similar quantitative expression

We created four yeast strains in which single TFs were epitope-tagged to facilitate ChIP. Cbf1, Gcn4, Met31 and Nrg1 were each tagged by creating in-frame fusions to the C-myc-Avi epitope tag (49) at the native chromosomal locus of each TF. Each strain also contained the bacterial biotin ligase, BirA, integrated at the *LYS2* locus. In each one of these four strains, we then created a synthetic promoter library comprising binding sites for Cbf1, Gcn4, Met31/Met32 and Nrg1, as described in (35). The number of total and unique promoters for each library is reported in Table 1.

These strain libraries were grown for either ChIP or expression analysis in both glucose and AAS conditions. In general, the libraries showed similar expression distributions to each other in both growth conditions (Supplementary Figures S1 and S2), indicating that the C-myc-Avi-tag does not alter protein function. The exception was the Cbf1-tagged strain, which showed a Cbf1-site-dependent effect on expression (Supplementary Figures S1 and S3). In all thermodynamic modeling, we corrected for the effect of tagged Cbf1 by introducing a polymerase-Cbf1 interaction term specific to the tagged Cbf1, but enforcing the TF–DNA interaction term to be the same between the tagged and untagged versions of the protein.

Quantitative expression in AAS of many promoters with multiple Gcn4 sites could not be determined due to high expression exceeding the dynamic range of the flow cytometer. For modeling purposes, these expression values were treated as missing. A list of all promoters and their expression data is available in Supplementary Table S3.

### ChIP of synthetic promoter libraries shows reproducible quantitative signal

**Figure 1.**
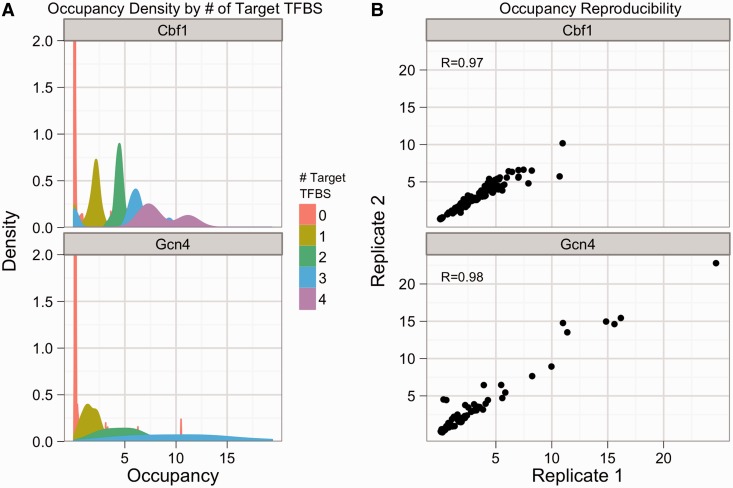
ChIP reveals quantitative differences in the occupancy of synthetic promoters by Cbf1 and Gcn4, and is highly reproducible. (**A**) The smoothed density of occupancy scores is shown for Cbf1 (top) and Gcn4 (bottom) for the AAS condition, colored by number of binding sites for the factor of interest. The y-axis has been cut at 2. (**B**) The relative occupancy for one ChIP replicate is plotted against the relative occupancy for another replicate for avi-tagged Cbf1 (top) and Gcn4 (bottom) in the AAS condition.

We first attempted to measure the occupancy of the tagged TFs on the promoters in our libraries. We performed three biological replicates of ChIP on each of the four strain libraries. We validated that our ChIP protocol was working by performing qPCR on known genomic targets of each of the four tagged TFs (Supplementary Figures S4 and S5). Samples from the Met31- and Nrg1-tagged libraries did not show target-specific enrichment (Supplementary Figure S5) and were excluded from all further analyses. Samples from the Cbf1- and Gcn4-tagged libraries were analyzed by high-throughput sequencing of the synthetic promoters in both the input and precipitated fractions (‘Methods’ section), which produces a measure of occupancy of each tagged TF on each member of the promoter libraries. The occupancy of Cbf1-containing promoters by Cbf1 increases almost linearly with the number of Cbf1 binding sites, with tight distributions around the median occupancy scores for a given number of Cbf1 sites (Figure 1A, left). Median Gcn4 occupancy also increases as a function of the number of Gcn4 binding sites, but in contrast with Cbf1, there is a wider dispersion of occupancy scores for different promoters with the same number of Gcn4 sites, suggesting that the context in which binding sites appear has a greater impact on Gcn4 than Cbf1. These occupancy distributions were highly reproducible across ChIP replicates (Figure 1B). These results suggest that we are obtaining accurate measures of TF occupancy across the synthetic promoters in our libraries.

### Thermodynamic modeling of expression shows good agreement between predicted and observed expression and occupancy

We used a thermodynamic model to analyze the data we collected from our synthetic promoter libraries. The thermodynamic model describes the expression of promoters in terms of the free energies of interaction (ΔG) of TFs with DNA, TFs with other TFs and TFs with RNA polymerase (51). In the thermodynamic model, each promoter is a collection of states, where each state is a particular configuration of bound and unbound proteins on the DNA. Each state is assigned a statistical weight based on the ΔGs of the specific interactions that occur in that particular state. The statistical weight of a state is the probability of observing the promoter in that particular state. The statistical weights are used to compute the fractional occupancy of RNA polymerase, which determines the expression level of the promoter. The fractional occupancy of RNA polymerase is computed as the sum of the weights of the promoter states in which polymerase is bound divided by the sum of the weights of all possible states. Thus, with values for the ΔGs of the interactions in the model, we can compute the occupancy of RNA polymerase on each promoter and provide a prediction of the expression level of that promoter.

We fit the model to data from synthetic promoter libraries by finding values for the ΔGs that minimize the difference between the predicted and experimentally measured expression values, across the entire library. During fitting, we always compare models that include different numbers and types of interactions. The resulting collection of ΔGs that comprise the best-fit model provides a quantitative description of the *cis*-regulatory interactions that govern the promoters in our libraries.

**Figure 2.**
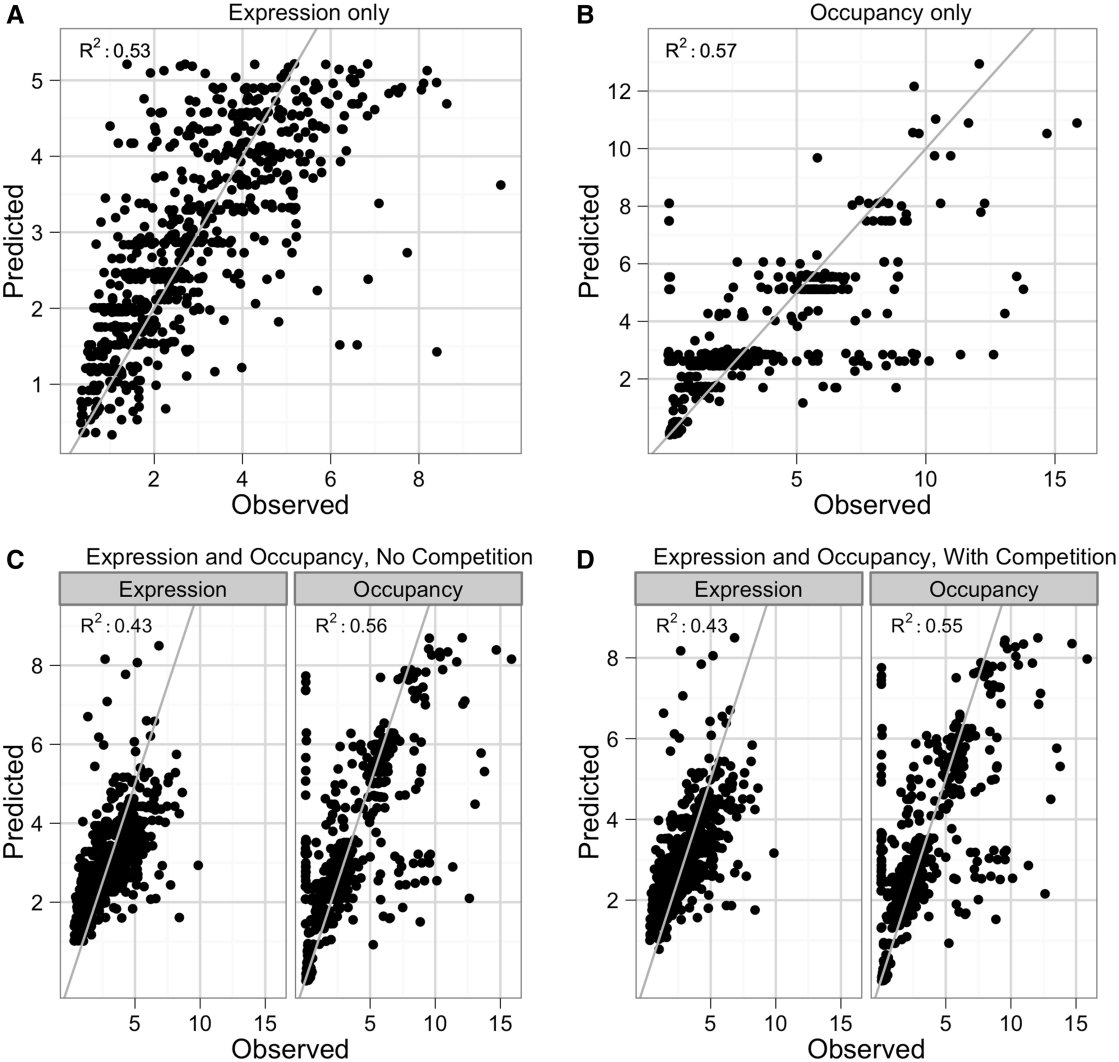
Fits of expression and occupancy by thermodynamic models. Observed data versus model-predicted values for thermodynamic models fit on (**A**) expression data, (**B**) occupancy data, (**C**) expression and occupancy data and (**D**) expression and occupancy data with Gcn4 competition. Gray line: best fit line.

We first fit the model using expression data collected in both glucose and AAS, ignoring the occupancy data (Figure 2A). The model uses the sequence composition of synthetic promoters to predict their expression levels. The overall fit was good (*R^2^* = 0.53) and comparable with the fit previously obtained by (36) (*R^2^* = 0.60), despite using two fewer parameters to model the data, having two different tagged TFs and having a greater diversity of promoters (212 unique promoters in glucose versus 131 unique promoters published previously). We performed 5-fold cross-validation on the final model and observed no loss of predictive power, suggesting that the model is not over-fit (Supplementary Table S4). The final parameter values for this model are listed in Supplementary Table S5. Notably, when fitting solely with expression data, no TF–TF interactions were found to significantly improve the fit.

We next attempted to fit the thermodynamic model using the normalized relative occupancy data we obtained from ChIP, ignoring the expression data. We used occupancy data collected from the Cbf1 and Gcn4-tagged strains grown in both the glucose and AAS conditions. We used these data to fit a thermodynamic model relating promoter sequence to the occupancy of the TFs on the synthetic promoters (Figure 2B). For this implementation, we considered only specific TF–DNA interactions; promoters with no specific binding sites for the tagged factors were excluded from the fit. The resulting model had a good fit to the data (*R^2^* = 0.54), suggesting that a large amount of TF occupancy can be accurately predicted by only the presence and configuration of TFBS. Cbf1 had no significant interaction terms with any other factors, which agrees with the linear increase of Cbf1 occupancy we observed with increasing numbers of Cbf1 sites. However, the ΔG_Gcn4__–__Gcn4_ and the ΔG_Gcn4__–__Nrg1_ interactions both made significant improvements to the fit of the model (*R^2^* = 0.56, *P* = 1.11e^−^^04^ and *R^2^* = 0.56, *P* = 5.20e^−^^05^, respectively, *F*-test with Bonferroni correction). Neither of these interactions was significant in the model that used only expression data, which suggests that the occupancy data contain extra information that reveals interactions that are undetectable in the expression data. Adding the ΔG_Gcn4__-__Gcn4_ interaction to a model that includes the ΔG_Gcn4__-__Nrg1_ interaction also led to a significant improvement in the performance of the model (*R^2^* = 0.57, *P* = 1.43e^−^^05^, *F*-test with Bonferroni correction). The final model, which includes the TF–DNA binding energies, along with the ΔG_Gcn4__-__Nrg1_ and ΔG_Gcn4__-__Gcn4_ interactions, predicts virtually no change in the DNA binding energy of Cbf1 between the two conditions (ΔΔG: −0.08), versus a large change in the DNA binding energy of Gcn4 when moving from glucose to AAS (ΔΔG: −2.74). This is consistent with the known regulation of Gcn4; amino acid starvation increases translation and transcription of Gcn4 mRNA, and stabilization of Gcn4p through dephosphorylation (52), all of which serve to increase the concentration of Gcn4p and decrease the free energy of binding. The final model resulted in a fit with explanatory power on par with the thermodynamic model of expression (*R^2^* = 0.57 for occupancy versus *R^2^* = 0.53 for expression), suggesting that the model describes the variation in both types of data equally well.

Finally, we attempted to fit a thermodynamic model with both expression and occupancy data simultaneously. We chose parameters to include based on which parameters were significant in the expression-only and occupancy-only fits (Supplementary Table S5). In general, the model converged on reasonable predictions of both expression and occupancy (Figure 2C). In particular, the model fit with both expression and occupancy data predicted both categories of data better than models fit separately to either source of data. The model fit only on occupancy data was incapable of predicting expression, as the ΔG_TF__–__RNAP_ terms could not be fit, and the model fit only on expression data predicted occupancy with an *R*^2^ of 0.36. In contrast, the model fit on both data types predicted expression with an *R*^2^ of 0.43 and occupancy with an *R*^2^ of 0.56 (Supplementary Table S4).

### Gcn4 site shows switching behavior

**Figure 3.**
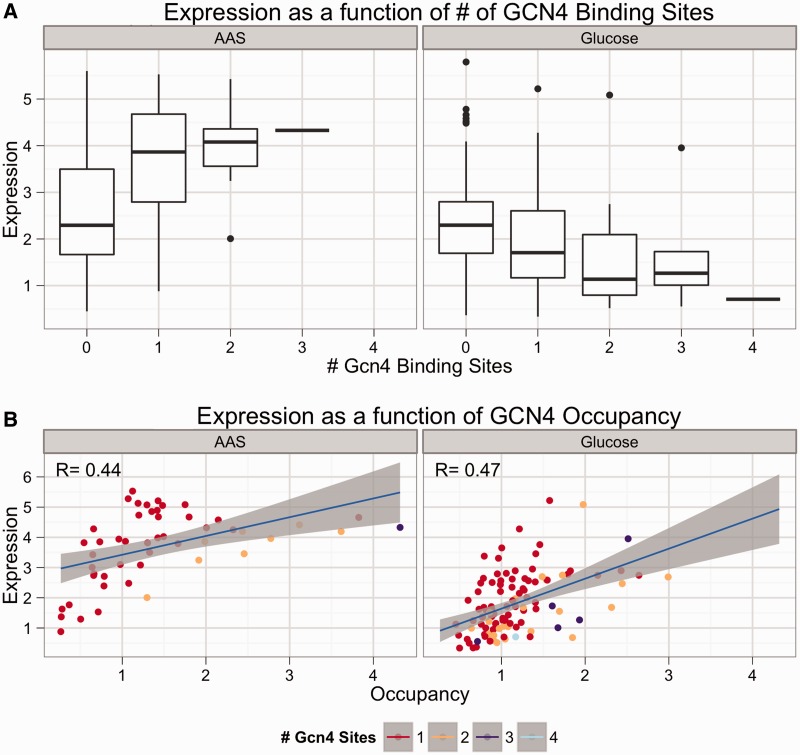
Gcn4 site activates in AAS and represses in glucose, but Gcn4 TF is an activator in both conditions. Strains bearing synthetic promoters with avi-tagged Gcn4 were grown as described in Methods in glucose and AAS media. Expression was measured via flow cytometry. Occupancy was measured by ChIP. (**A**) A boxplot of expression of promoters in AAS (left) and glucose (right) grouped by the number of Gcn4 sites present in the promoter shows that Gcn4 sites repress weakly in glucose but activate strongly in AAS. (**B**) A plot of expression versus total Gcn4 occupancy in AAS (left) and glucose (right) shows that Gcn4 occupancy correlates positively with expression in both AAS and glucose. This suggests a repressive factor is binding the Gcn4 site in glucose in competition with Gcn4. There are fewer points in AAS than in glucose due to the strong activating effect of multiple Gcn4 sites in AAS.

The Gcn4 binding site showed different behavior between the glucose and AAS conditions. Figure 3A shows the aggregate effect on expression of increasing the number of Gcn4 sites in promoters with many different combinations of Cbf1, Nrg1 and Met31/32 binding sites. In AAS, the Gcn4 binding site was a strong activating sequence (Figure 3A, left) regardless of which other sites were present, consistent with the known role of Gcn4 in recruiting mediator and other transcriptional complexes (53,54) in response to limiting amounts of amino acids. In contrast, the Gcn4 site functioned as a weak repressor when cells were grown in glucose (Figure 3A, right) in many different promoter contexts. The switching behavior occurred regardless of which factor was tagged (data not shown), indicating that the repressive effect in glucose is independent of the epitope tag. When modeling only expression, allowing the Gcn4-RNAP interaction to differ between conditions revealed the same trend; the site activates in AAS conditions but represses weakly in glucose (Supplementary Table S5). Forcing the model to use the same polymerase interaction term for the Gcn4 site in both conditions resulted in a significantly worse fit (*R^2^* = 0.53 versus 0.43, *P* < 10^−^^16^, *F*-test). Attempting to fit the model by constraining the ΔG_Gcn4__–__RNAP_ term while allowing the ΔG_Gcn4__−__DNA_ in glucose to vary, as was done previously (36), resulted in a good fit (*R^2^* = 0.50), but the resulting change in binding energy equates to 8.12 × 10^−^^14^-fold lower apparent Ka. This change in binding energy is not biologically reasonable and is likely an artifact of fitting the data to an inappropriate model. Taken together, the results suggest that the switching behavior of Gcn4 is not a result of differential affinity of Gcn4 for its binding site between the glucose and AAS conditions.

There are two possibilities for why the Gcn4 site switches behavior between conditions. The first is that post-translational modifications, or interactions with other proteins, cause Gcn4 to convert between two forms, an activating form that predominates in AAS and a repressing form that predominates in glucose. Alternatively, Gcn4 may always be an activator, but in glucose competes with a repressor for binding to the same site. The occupancy data can distinguish between these two hypotheses. If expression is negatively correlated with Gcn4 occupancy in glucose, it would suggest that Gcn4 is switching from an activator to a repressor in glucose. However, if expression is positively correlated with Gcn4 occupancy in glucose, it would suggest that Gcn4 is still an activator in glucose and that a repressive factor competes with Gcn4 for binding in glucose. To distinguish between these two hypotheses, we plotted Gcn4 occupancy versus expression for both the glucose and AAS conditions (Figure 3B). As expected, there is a strong positive correlation between Gcn4 occupancy and expression in AAS (Figure 3B, left), where Gcn4 is known to be a transcriptional activator. However, there is also a positive correlation between Gcn4 occupancy and expression in glucose (Figure 3B, right). This suggests that Gcn4 is still a transcriptional activator in glucose, and that the repressive effects of Gcn4 sites in glucose result from a repressive factor that competes with Gcn4 for binding to the Gcn4 site. The idea of a Gcn4 competitor has some experimental support. For instance, high concentrations of Gcn4 were shown to compete with an unidentified nuclear protein on the HIS4 promoter (44). Moreover, the factor Bas1 binds an overlapping motif with Gcn4 (55) and is suspected of competing with Gcn4 (56). Alternatively, nucleosomes may effectively compete for binding to the Gcn4 site in rich medium, where Gcn4 concentrations are lower. Thus, both our data and the literature support the idea of competitive binding occurring at the Gcn4 site.

### Competitive model of binding better explains Gcn4 expression and occupancy

We extended the thermodynamic model to incorporate competitive binding at the Gcn4 site between Gcn4 and an unknown repressor. We did this by adding promoter states where the competing protein is bound to the site instead of Gcn4. To fit the model, we assumed that the effect on polymerase of the two competitors was consistent between conditions, but allowed the relative affinity of the two factors for the Gcn4 site to vary between conditions. When this model was fit with only expression data, it performed exactly the same as the model that allows Gcn4 to switch from an activator to a repressor in different conditions. Thus, the switching behavior of the Gcn4 binding site can be modeled equally well by either having a repressive factor compete with Gcn4 for binding (competitive model) or by having the Gcn4 protein switch from an activator to a repressor between conditions (non-competitive model). With only expression data, these two models cannot be distinguished.

We determined whether fitting the models simultaneously to both expression data and occupancy data would distinguish between the competitive and non-competitive models. The combined expression and occupancy data were already fit to the non-competitive model (Figure 2C), and we performed the same analysis with the competitive model. Both models resulted in similar fits (expression, occupancy *R*^2^: 0.425, 0.556 and 0.431, 0.554, non-competitive and competitive models, respectively; see also Supplementary Table S5), although the competitive model is marginally better at predicting expression. However, the non-competitive model consistently set the glucose Gcn4-RNAP term as highly unfavorable (i.e. made Gcn4 a strong repressor in glucose) and resulted in fits that rarely converged to similar parameter values (<20% of the time) and usually resulted in singularities in the parameter Jacobian matrix (>90%). In contrast, the competitive model resulted in the same fit 50% of the time, with a non-singular Jacobian matrix. In this best fit, the difference in Gcn4–DNA binding energies between the two conditions equates to fold change in the apparent Ka of ∼25-fold, which is somewhat larger than previous estimates (57), but is still biophysically plausible. Thus, incorporating competition in the model resulted in a more biologically reasonable fit.

## DISCUSSION

We sought to improve our quantitative understanding of the biophysical mechanisms underlying *cis*-regulation by incorporating ChIP data into existing statistical thermodynamic models of regulation. We compared models parameterized with only expression, only occupancy data and with both types of data. Comparing the results of these modeling procedures revealed several interesting features.

Gcn4 occupancy was more sensitive to the particular configuration of binding sites in promoters, whereas Cbf1 appeared to bind promoters with almost no context-dependent effects. In light of this result, it is interesting to note that Cbf1 recruits chromatin remodeling complexes (58,59), whereas Gcn4 directly recruits the transcriptional machinery (53,54). Proper Cbf1 function may require the ability to bind DNA regardless of what other factors are binding nearby, including nucleosomes. In contrast, having Gcn4 occupancy depend on a more specific local sequence context might prevent inappropriate activation of non-target genes. This line of reasoning suggests that TFs, which directly recruit polymerase and related subunits, will be more heavily influenced by binding site context than so-called pioneer factors (60), which are involved in earlier processes, such as chromatin remodeling.

Modeling complex systems requires a balance between sufficient model complexity to capture the observed trends and sufficient simplicity to avoid over-fitting the data. We have attempted to err on the side of simplicity. Although the model generally performed well, the predictive ability with regards to expression decreased when incorporating the occupancy data because the fitting tended to favor fitting the occupancy data. This is partially due to the magnitude of residuals in the occupancy data relative to the residuals in the expression data. But it also suggests that the relationship between occupancy and expression in the data is more complex than the simple protein–protein interaction energies expressed in our model. Additionally, in our attempt to keep the model as simple as possible, we have avoided incorporating spacing, orientation and edge effects. Subsequent model refinements could look at these effects and attempt to describe a richer activation scheme than our current model.

In all, we find that integrating protein binding information in the form of ChIP data with expression data provides the ability to quantitatively reason about the biophysical mechanisms that underlie observed expression data and to distinguish between distinct biophysical mechanisms that can give rise to the same expression patterns.

## SUPPLEMENTARY DATA

Supplementary Data are available at NAR Online, including [61–65].

## FUNDING

National Institutes of Health (NIH) [RGM092910A to B.A.C., P30 CS91842 to the Siteman Cancer Center, UL1RR024992 to the Institute of Clinical and Translational Sciences (ICTS)]. Funding for open access charge: NIH [RGM092910A].

*Conflict of interest statement*. None declared.

## Supplementary Material

Supplementary Data
