# Supplementary material for: Discrimination between thermodynamic models of cis-regulation using transcription factor occupancy data
Source: Nucleic Acids Res. 2013 Nov 27;42(4):2224–34. doi: 10.1093/nar/gkt1230 (PMC3936720; doi:10.1093/nar/gkt1230)
Supplement: Supplementary Data [file supp_42_4_2224__index.html]

Discrimination between thermodynamic models of cis-regulation using transcription factor occupancy data — Discrimination between thermodynamic models of cis-regulation using transcription factor occupancy data — Supplementary Data 

# Discrimination between thermodynamic models of *cis*-regulation using transcription factor occupancy data

## Supplementary Data

files

**Files in this Data Supplement:**

- Supplementary Data - pdf file
