# Supplementary material for: Discrimination between thermodynamic models of cis-regulation using transcription factor occupancy data
Source: Nucleic Acids Res. 2013 Nov 27;42(4):2224–34. doi: 10.1093/nar/gkt1230 (PMC3936720; doi:10.1093/nar/gkt1230)
Supplement: Supplementary Data [file supp_gkt1230_nar-01662-m-2013-File005.pdf]

Supplemental Methods

Library Sequencing

Synthetic promoters were sequenced on the Illumina MiSeq platform using a double barcoding strategy. One of 96-well-specific barcoded primers was used with one of forty plate-specific barcoded primers to colony PCR the synthetic promoters such that each promoter was amplified with a unique combination of well and plate primers (well and plate primers are listed in supplementary table S1). The well primers included a SalI restriction site and the plate primers included an Mfe1 restriction site at the 3' end. Five uL of each PCR reaction was pooled together and ethanol precipitated, resuspended in 10 mLs of water, phenol/chloroform extracted, then ethanol precipitated and resuspended in 1 mL of H2O. The DNA was size-selected (150bp to 800bp) on a 1.5% TAE gel and purified, then ligated to annealed oligos RZ231 and RZ233 in the presence of EcoR1-HF (NEB R3101) and Mfe1 (NEB R0589S) and purified. The purified material was then ligated to annealed oligos RZ230 and RZ232 in the presence of Xho1 (NEB R0146S) and Sal1-HF (NEB R3138), then size selected (150-700 bp) on a 1.5% TAE Agarose gel . The library was then sequenced on the MiSeq platform using 250 cycles for read one and 50 cycles for read two. The resulting sequence data was analyzed by custom python scripts that using a minimum hamming distance approach to determine the TFBS composition of the promoters and the originating well and plate of the promoter.

Biotin-ChIP

One liter of pooled culture growth was incubated with 1% final concentration of formaldehyde for 15 minutes at room temperature, followed by addition of 150 mLs of 2.5M glycine for five minutes. The cultures were centrifuged, then washed three times with chilled TBS. The cell pellet was frozen at least overnight at -80C. Frozen pellets were thawed on ice and resuspended in 2 mLs of Lysis Buffer (50 mM HEPES, 150 mM NaCl, 1 mM EDTA, 1% v/v Triton X-100, 0.1% w/v sodium deoxycholate, 0.1% w/v SDS) and protease inhibitor (Roche #11836170001). Each replicate bead beat using zirconium silicate beads using a Biospec Products Mini Bead Beater six times for three minutes each time with a minute in ice-water between beatings. The lysed material was extracted by centrifugation and the pellets resuspended in a final volume of 5 mLs of Lysis Buffer in a 15 mL centrifuge tube. The resuspended material was sonicated twice for 30 seconds with a Branson Sonifier 250 tip sonicator at power level six, duty 75% followed by four times for 30 seconds at power level five, duty 75%, with at least two minutes on ice between sonications. The supernatant was clarified by centrifugation, then applied to 500 uL PBS-washed Dynal M280 streptavidin-coated magnetic beads (Life Technologies, 112-05D) and incubated at room temperature for one hour. The beads were bound to magnets and the supernatant removed and set aside as "input" (IN) material. The beads were washed twice for five minutes per wash in each of Lysis Buffer, High Salt Lysis Buffer (50 mM HEPES, 0.5M NaCl, 1 mM EDTA, 1% v/v Triton X-100, 0.1% w/v sodium deoxycholate), LiCl Wash Buffer (500 mM LiCl, 1% NP-40 alternative, 10 mM Tris pH 8.0, 1 mM EDTA), SDS Wash Buffer (10 mM Tris pH 8.0, 1 mM EDTA, 3% SDS), and TE (10 mM Tris pH 8.0, 1 mM EDTA). The beads were resuspended in 250 uL TE + 0.5% SDS + 10 uL of 20 mg/mL Proteinase-K (NEB P8102S) and distributed into three 250 uL PCR tubes per replicate. Then 72.5 uL of IN material was

combined with 72.5 uL of TE + 1% SDS to which 10 uL of 20 mg/mL Proteinase-K was added and distributed into three 250 uL PCR tubes per replicate. The tubes were incubated for four hours at 42C, two hours at 72C, and six hours at 65C. The material from each replicate was recombined and purified via ChIP cleanup columns (Zymo D5205), eluting in 40 uL of elution buffer.

Thermodynamic Model of Transcription

Modeling of expression and occupancy used the thermodyamic model of transcription described previously (28,34,35). The model considers unbound DNA as a reference state and computes the statistical weight of each possible configuration *k* of transcription factors and proteins bound to the DNA as:

$$W_k = e^{-\Delta G_k}$$

Where ΔG_k is given as:

$$\Delta G_k = \sum_{i=1}^{L} (\Delta G_{tf\,i\,DNA} + \Delta G_{tf\,i,RNAP} \cdot \delta(RNAP)) \cdot \delta(TF_i) + \sum_{i=1}^{L-1} \sum_{j=i+1}^{L} \Delta G_{ixn\,tf\,i,j} \cdot \delta(TF_i) \cdot \delta(TF_j) \cdot \varepsilon(i,j)$$

where L is the number of TF binding sites in the synthetic promoter, $\Delta G_{tf\,i\,DNA}$ is the binding energy of the TF at site i, reflecting its concentration and affinity for the site, $\Delta G_{tf\,i\,RNAP}$ is the binding energy between the TF at site i and RNAP, δ(*RNAP*) is one if RNAP is bound in the current state and zero otherwise, $\Delta G_{ixn\,tf\,i,j}$ is the binding energy between the TF at site i and the TF at site j, $\delta(TF_x)$ is one if the TF at site x is bound in the current state and zero otherwise, and ε(i,j) is one if there are no other TFs bound between sites i and j in the current state, and zero otherwise. The probability of polymerase bound is then given as:

$$P(RNAP_{bound}) = \frac{\sum_{k=1}^{N} W_k \delta_k(RNAP)}{\sum_{k=1}^{N} W_k}$$

Where N is the total number of states (2^L for non-competitive binding), and $\delta_k(RNAP)$ is one if RNAP is bound in state k and zero otherwise. The probability of occupancy for a particular TF is computed as:

$$P(\geq 1\ TF_{bound}) = \frac{\sum_{k=1}^{N} w_k \delta_k(TF)}{\sum_{k=1}^{N} w_k}$$

Where N is the total number of states (2^L for non-competitive binding), and δ_k(TF) is one if the TF is bound to one or more of its sites in the state and zero otherwise. The observed occupancy and expression values were assumed to be linearly related to the predicted probabilities, respectively:

$$Occupancy = \alpha \cdot P(\geq 1\ TF_{bound})$$

And

$$\mathrm{E}xpression = \beta \cdot P(RNAP_{bound})$$

Where α and β are the least-squares estimates. The current model does not account for non-specific TF-DNA interactions and always predicts an occupancy of zero for promoters with no specific binding site for the factor of interest. Therefore, promoters lacking a specific binding site for the factor whose occupancy was being calculated were excluded from model estimation and validation. Model parameters were recovered in several ways. First, by performing simultaneous optimization with only expression data. Second by fitting with occupancy data. Third, by fitting to occupancy and expression data. When fitting only to occupancy data, TF-DNA and TF-TF binding energies were explored with a simultaneous fit to all environments. When fitting to expression data, the optimization was carried out simultaneously for multiple environments and factors largely as previously described (35) with modifications as follows. First, expression values for multiple biological replicates within a particular strain background were collapsed into a single promoter using the median expression of all biological replicates. Second, no down-weighting of short-promoter residuals was used. Finally, the optimization was done in R using nlminb with default parameters. When fitting with both expression and occupancy data, the occupancy data was first re-scaled by the ratio of the means of the occupancy and expression data to put it on a similar quantitative scale to the expression data to ensure that neither the occupancy nor the expression would dominate the residual sum of squares for fitting. Optimization was performed as for fitting with expression data, with the probability of both occupancy and expression scaled to the mean of all observed values.

qPCR of ChIP Samples

Factor-specific qPCR primers were chosen by selecting the most highly enriched probes for the factor from Harbison, *et al.* (61), scanning the probe sequence with Patser (62) for motif matches using motifs from Zhao and Stormo (63) for Cbf1 and from Spivak and Stormo (64) for the remaining factors, then using Primer3 to design qPCR primers that flanked the best motif matches. SUC2 was used as an internal control region. Briefly, qPCR was carried out using three independent dilutions of two ChIP

replicates for both IP and IN samples (0.004 - 0.04 ng/uL final concentration) using SYBR Green QPCR Master Mix (Thermo Scientific AB-1158/A) for detection in a final volume of 25 uL, with qPCR primers (table 3.6) at a final concentration of 0.3uM. The qPCR was run on a Stratagene Mx3000p thermocycler. Replicates were averaged and analyzed using the $\Delta\Delta$Ct method.

Figure S1: Expression distributions in glucose similar across all libraries except Cbf1

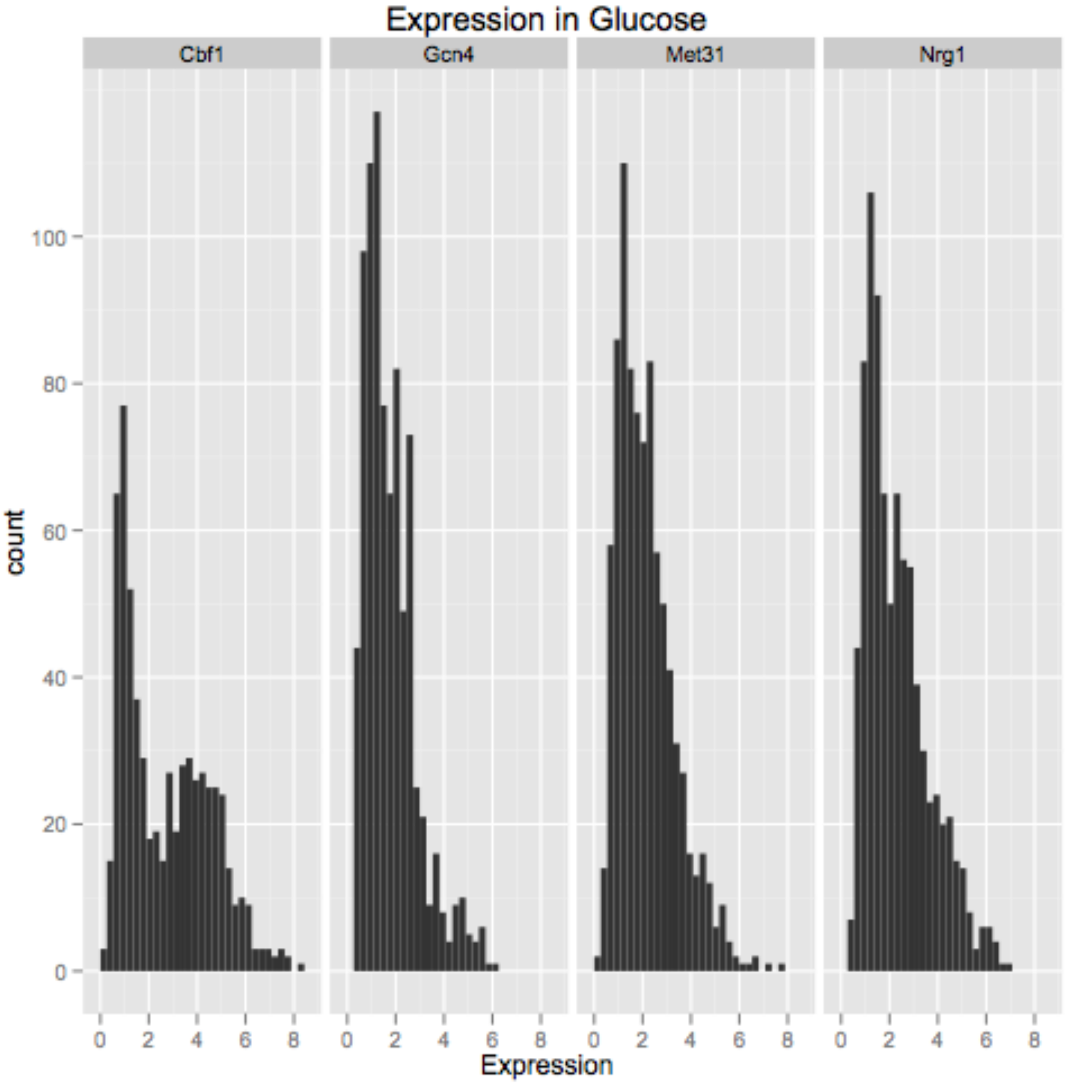

Libraries were grown to mid-log phase in SCB-Ura, fixed with a final concentration of 1% formaldehyde, and the fluorescence intensities measured by flow cytometry. The distribution of all libraries is similar except for Cbf1 (after multiple hypothesis correction, $P < 10^{-16}$ for Cbf1-Gcn4, Cbf1-Met31, Cbf1-Nrg1; $P=0.18$, Gcn4-Met31; $P=0.06$, Gcn4-Nrg1; $P=0.45$, Met31-Nrg1, Kolmogorov-Smirnov test).

Figure S2: Expression distributions in AAS similar across all libraries

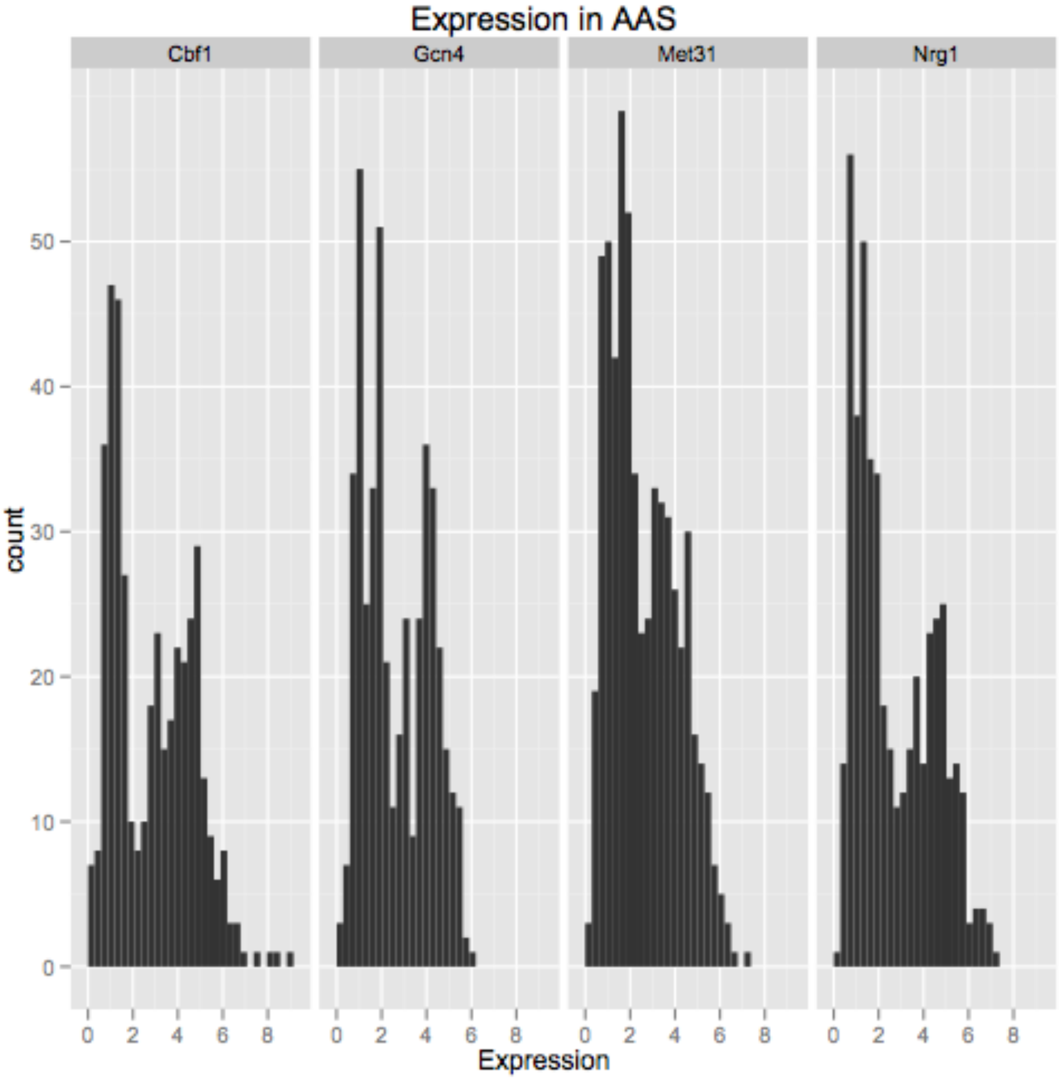

Libraries were grown to mid-log phase in SCB-Ura, fixed with a final concentration of 1% formaldehyde, and the fluorescence intensities measured by flow cytometry. The distribution of all libraries is similar but with some significant differences by Kolmogorov-Smirnov testing, suggesting some strain-specific effects in AAS, probably due to the protein tag (after multiple hypothesis correction, P = 0.0096, Cbf1-Gcn4; P=0.20, Cbf1-Met31; P=0.00017, Cbf1-Nrg1; P=0.41 Gcn4-Met31; P=$6.3e^{-10}$, Gcn4-Nrg1; P=$1.3e^{-5}$ Met31-Nrg1).

Figure S3: Avi-tagging increases Cbf1 activation potential, but not Gcn4

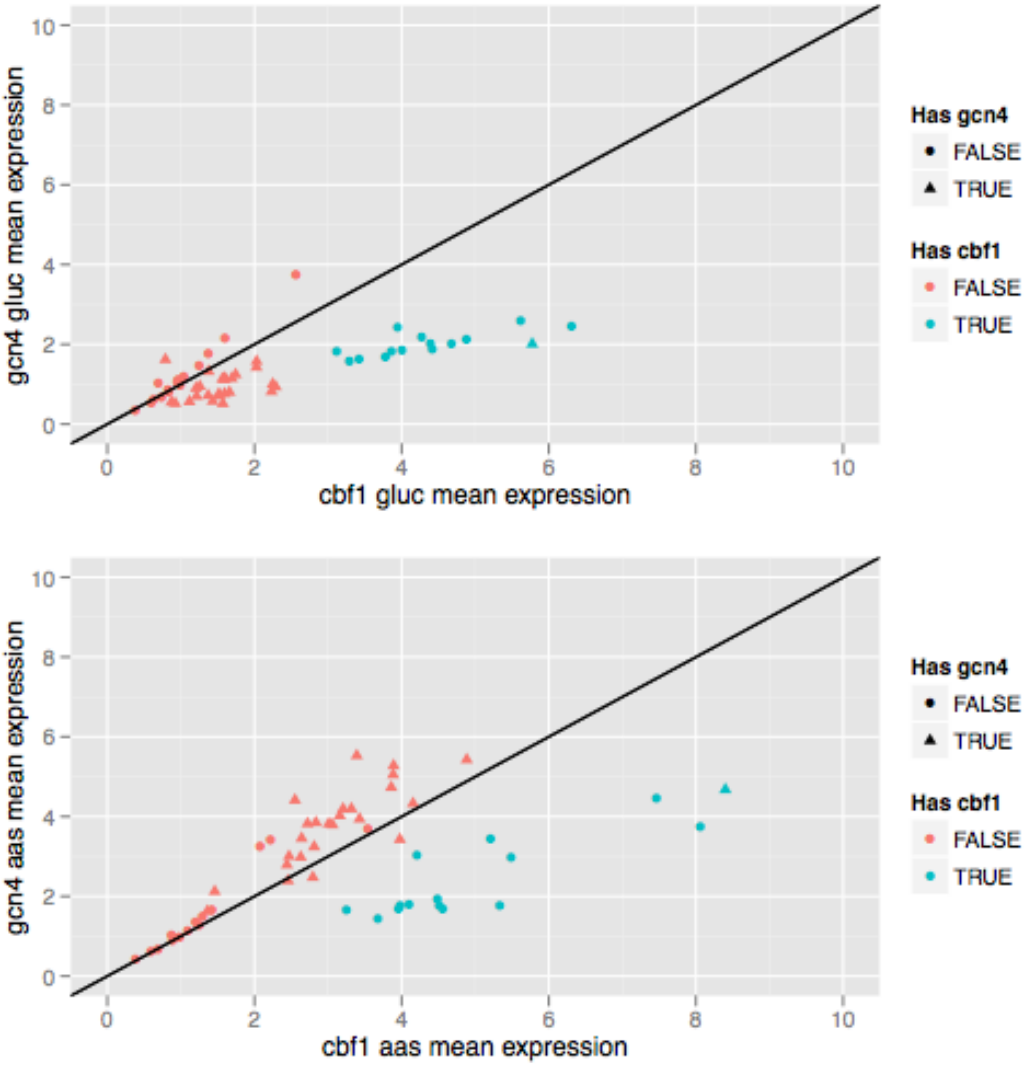

Mean expression of identical promoters in the Cbf1-tagged background and Gcn4-tagged background were compared. Promoters without cbf1 in them (red) generally show good agreement between the two libraries, falling along the black line. Promoters with Cbf1 sites in them (blue) consistently show higher expression in the Cbf1-tagged background than in the Gcn4-tagged background. Promoters with Gcn4 sites in them (triangles) do not appear to differ between the two libraries, indicating that the tag on Gcn4 has little if any effect on expression.

Figure S4: Specific enrichment of bound regions for Cbf1 and Gcn4 in glucose and AAS

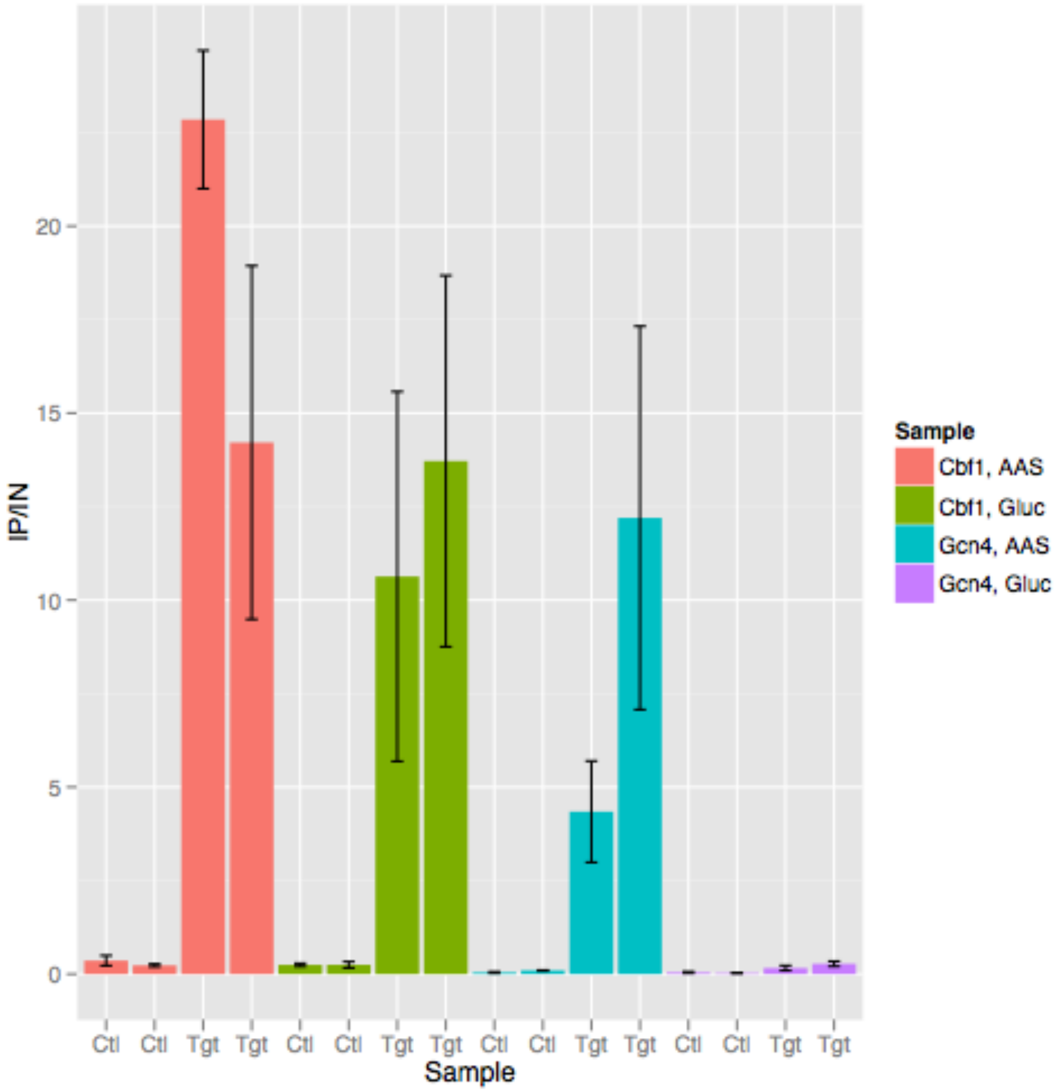

ChIP was performed on synthetic promoter-bearing strains with Avi-tagged Cbf1 and avi-tagged Gcn4 as outlined in methods. Enrichment was gauged by qPCR of a control (Ctl) region (SUC2) versus a target (Tgt) region (Ade 3 for Cbf1, CPA2 for Gcn4). Gluc is in Glucose, AAS is in Amino Acid Starvation. The target region is differentially bound with respect to the control region for both factors in all conditions, though only marginally so for Gcn4 in Glucose. The low enrichment of Gcn4 in Glucose is expected due to low functional expression of Gcn4 in that condition, based on Western Blotting (not shown) and previous literature [REF].

Figure S5: Limited or no specific enrichment of Met31 and Nrg1 ChIP

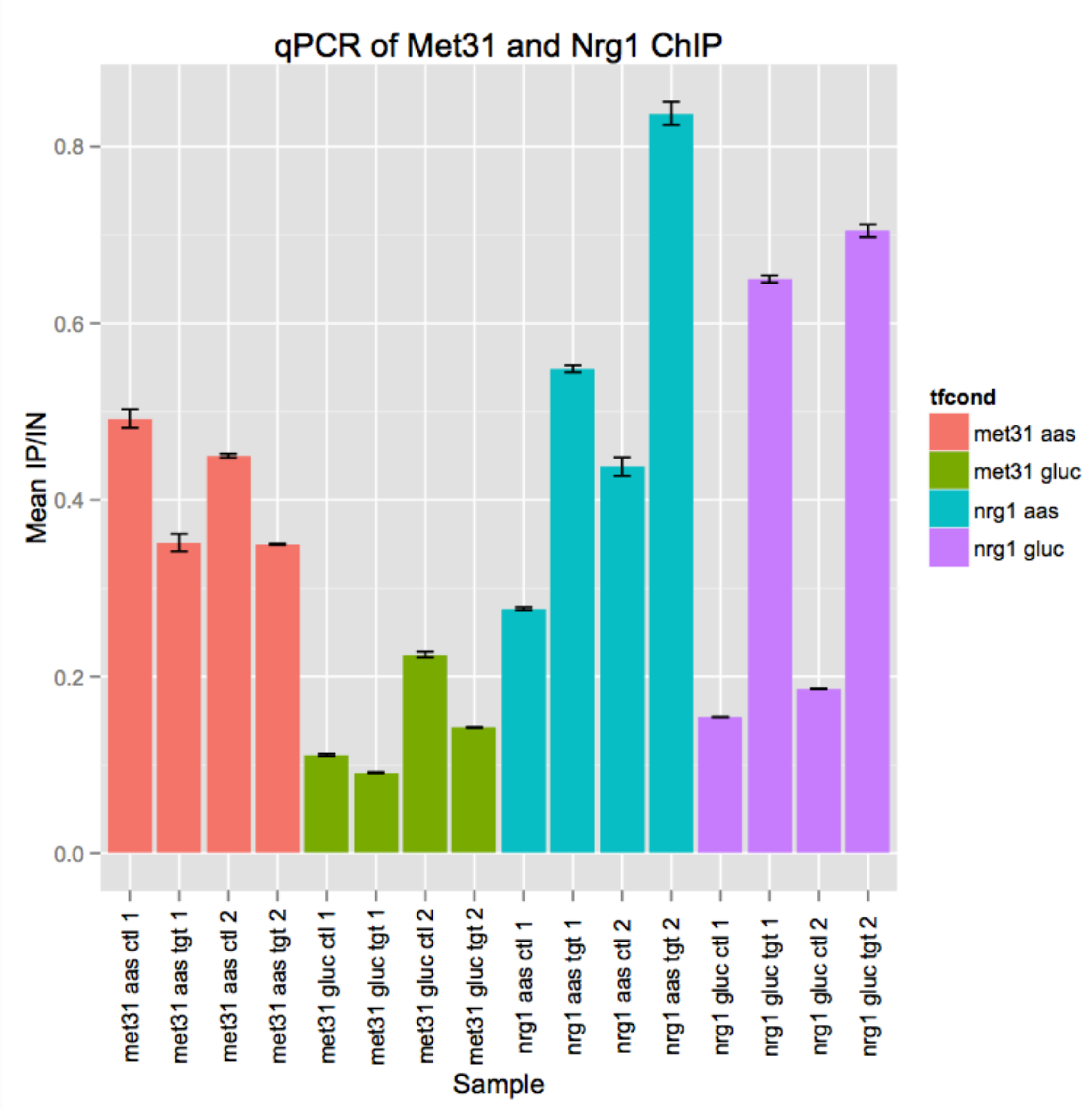

ChIP was performed on synthetic promoter-bearing strains with Avi-tagged Met31 and avi-tagged Nrg1 as outlined in methods. Enrichment was gauged by qPCR of a control (Ctl) region (SUC2) versus a target (Tgt) region (CAF120 for Met31, NRG1 for Nrg1). Gluc is in Glucose, AAS is in Amino Acid Starvation. The target region is not differentially bound with respect to the control region for Met31. Although Nrg1 looks significantly enriched, the level of enrichment is similar to levels obtained from ChIP done in strains with only BirA (data not shown).

Table S1: Barcoded “well” and “plate” PCR primers used for library sequencing.

Sequence	Barcode	Use
CTACGTCGACACACACTTAATCGTTCTTCCACACGGATC	ACACACT	WA1[a]
CTACGTCGACACACTACTAATCGTTCTTCCACACGGATC	ACACTAC	WA2[a]
CTACGTCGACACAGATGTAATCGTTCTTCCACACGGATC	ACAGATG	WA3[a]
CTACGTCGACACATCGTTAATCGTTCTTCCACACGGATC	ACATCGT	WA4[a], CLP1[b]
CTACGTCGACACATGAGTAATCGTTCTTCCACACGGATC	ACATGAG	WA5[a], CLP2[b]
CTACGTCGACACGAGACTAATCGTTCTTCCACACGGATC	ACGAGAC	WA6[a], CLP3[b]
CTACGTCGACACGTCTGTAATCGTTCTTCCACACGGATC	ACGTCTG	WA7[a], CAP1[b]
CTACGTCGACACTACTATAATCGTTCTTCCACACGGATC	ACTACTA	WA8[a], CAP2[b]
CTACGTCGACACTAGCTTAATCGTTCTTCCACACGGATC	ACTAGCT	WA9[a], CAP3[b]
CTACGTCGACACTATGCTAATCGTTCTTCCACACGGATC	ACTATGC	WA10[a], GLP1[b]
CTACGTCGACACTGAGATAATCGTTCTTCCACACGGATC	ACTGAGA	WA11[a], GLP2[b]
CTACGTCGACACTGCATTAATCGTTCTTCCACACGGATC	ACTGCAT	WA12[a]
CTACGTCGACAGACAGCTAATCGTTCTTCCACACGGATC	AGACAGC	WB1[a]
CTACGTCGACAGAGCACTAATCGTTCTTCCACACGGATC	AGAGCAC	WB2[a]
CTACGTCGACAGATGCATAATCGTTCTTCCACACGGATC	AGATGCA	WB3[a]
CTACGTCGACAGCAGCGTAATCGTTCTTCCACACGGATC	AGCAGCG	WB4[a], GLP3[b]
CTACGTCGACAGCATGATAATCGTTCTTCCACACGGATC	AGCATGA	WB5[a], GAP1
CTACGTCGACAGCGAGTTAATCGTTCTTCCACACGGATC	AGCGAGT	WB6[a], GAP2[b]

Sequence	Barcode	Use
CTACGTCGACAGCTATCTAATCGTTCTTCCACACGGATC	AGCTATC	WB7[a], GAP3[b]
CTACGTCGACAGCTCATTAATCGTTCTTCCACACGGATC	AGCTCAT	WB8[a], MLP1[b]
CTACGTCGACAGTACAGTAATCGTTCTTCCACACGGATC	AGTACAG	WB9[a], MLP2[b]
CTACGTCGACATAGCGATAATCGTTCTTCCACACGGATC	ATAGCGA	WB10[a], MLP3[b]
CTACGTCGACATCACACTAATCGTTCTTCCACACGGATC	ATCACAC	WB11[a], MAP1[b]
CTACGTCGACATCTACATAATCGTTCTTCCACACGGATC	ATCTACA	WB12[a], MAP2[b]
CTACGTCGACATGCAGACTAATCGTTCTTCCACACGGATC	ATGCAGAC	WC1[a]
CTACGTCGACATGCTCGCTAATCGTTCTTCCACACGGATC	ATGCTCGC	WC2[a]
CTACGTCGACCACACATCTAATCGTTCTTCCACACGGATC	CACACATC	WC3[a]
CTACGTCGACCACTACGCTAATCGTTCTTCCACACGGATC	CACTACGC	WC4[a], GAK3[b]
CTACGTCGACCACTCTCCTAATCGTTCTTCCACACGGATC	CACTCTCC	WC5[a]
CTACGTCGACCAGATAGCTAATCGTTCTTCCACACGGATC	CAGATAGC	WC6[a]
CTACGTCGACCAGCGCTCTAATCGTTCTTCCACACGGATC	CAGCGCTC	WC7[a]
CTACGTCGACCATATCACTAATCGTTCTTCCACACGGATC	CATATCAC	WC8[a]
CTACGTCGACCATGATCCTAATCGTTCTTCCACACGGATC	CATGATCC	WC9[a]
CTACGTCGACCGACGAGCTAATCGTTCTTCCACACGGATC	CGACGAGC	WC10[a], MAP3[b]
CTACGTCGACCGAGACGCTAATCGTTCTTCCACACGGATC	CGAGACGC	WC11[a], NLP1[b]
CTACGTCGACCGATAGACTAATCGTTCTTCCACACGGATC	CGATAGAC	WC12[a]
CTACGTCGACCGCGCTGCTAATCGTTCTTCCACACGGATC	CGCGCTGC	WD1[a]

Sequence	Barcode	Use
CTACGTCGACCGCTGACCTAATCGTTCTTCCACACGGATC	CGCTGACC	WD2[a]
CTACGTCGACCGTCACACTAATCGTTCTTCCACACGGATC	CGTCACAC	WD3[a]
CTACGTCGACCGTGTATCTAATCGTTCTTCCACACGGATC	CGTGTATC	WD4[a]
CTACGTCGACCTACAGTCTAATCGTTCTTCCACACGGATC	CTACAGTC	WD5[a]
CTACGTCGACCTAGCATCTAATCGTTCTTCCACACGGATC	CTAGCATC	WD6[a]
CTACGTCGACCTATATGCTAATCGTTCTTCCACACGGATC	CTATATGC	WD7[a]
CTACGTCGACCTCAGCACTAATCGTTCTTCCACACGGATC	CTCAGCAC	WD8[a]
CTACGTCGACCTCGAGCCTAATCGTTCTTCCACACGGATC	CTCGAGCC	WD9[a]
CTACGTCGACCTCGTAGCTAATCGTTCTTCCACACGGATC	CTCGTAGC	WD10[a], NLP2[b]
CTACGTCGACCTCTCGTCTAATCGTTCTTCCACACGGATC	CTCTCGTC	WD11[a], NLP3[b]
CTACGTCGACCTGACGCCTAATCGTTCTTCCACACGGATC	CTGACGCC	WD12[a], NAP1[b]
CTACGTCGACCTGCGACGCTAATCGTTCTTCCACACGGATC	CTGCGACGC	WE1[a]
CTACGTCGACCTGTCAGGCTAATCGTTCTTCCACACGGATC	CTGTCAGGC	WE2[a]
CTACGTCGACGACATCTGCTAATCGTTCTTCCACACGGATC	GACATCTGC	WE3[a]
CTACGTCGACGACGCGAGCTAATCGTTCTTCCACACGGATC	GACGCGAGC	WE4[a], NAP2[b]
CTACGTCGACGAGACACGCTAATCGTTCTTCCACACGGATC	GAGACACGC	WE5[a]
CTACGTCGACGAGCACGGCTAATCGTTCTTCCACACGGATC	GAGCACGGC	WE6[a]
CTACGTCGACGAGTAGCGCTAATCGTTCTTCCACACGGATC	GAGTAGCGC	WE7[a], NAP3[b]
CTACGTCGACGAGTGTAGCTAATCGTTCTTCCACACGGATC	GAGTGTAGC	WE8[a]
CTACGTCGACGATAGATGCTAATCGTTCTTCCACACGGATC	GATAGATGC	WE9[a]
CTACGTCGACGATCAGAGCTAATCGTTCTTCCACACGGATC	GATCAGAGC	WE10[a], CLK1[b]

Sequence	Barcode	Use
CTACGTCGACGATGTAGGCTAATCGTTCTTCCACACGGATC	GATGTAGGC	WE11[a], CLK2[b]
CTACGTCGACGCACTCAGCTAATCGTTCTTCCACACGGATC	GCACTCAGC	WE12[a], CLK3[b]
CTACGTCGACGCAGAGTGCTAATCGTTCTTCCACACGGATC	GCAGAGTGC	WF1[a]
CTACGTCGACGCAGCAGGCTAATCGTTCTTCCACACGGATC	GCAGCAGGC	WF2[a]
CTACGTCGACGCGACGAGCTAATCGTTCTTCCACACGGATC	GCGACGAGC	WF3[a], CAK1[b]
CTACGTCGACGCTCATGGCTAATCGTTCTTCCACACGGATC	GCTCATGGC	WF4[a], CAK2[b]
CTACGTCGACGCTCGACGCTAATCGTTCTTCCACACGGATC	GCTCGACGC	WF5[a], CAK3[b]
CTACGTCGACGTACATCGCTAATCGTTCTTCCACACGGATC	GTACATCGC	WF6[a]
CTACGTCGACGTAGACAGCTAATCGTTCTTCCACACGGATC	GTAGACAGC	WF7[a]
CTACGTCGACGTATCACGCTAATCGTTCTTCCACACGGATC	GTATCACGC	WF8[a]
CTACGTCGACGTCACTGGCTAATCGTTCTTCCACACGGATC	GTCACTGGC	WF9[a], GLK1[b]
CTACGTCGACGTCTGATGCTAATCGTTCTTCCACACGGATC	GTCTGATGC	WF10[a]
CTACGTCGACGTGAGCGGCTAATCGTTCTTCCACACGGATC	GTGAGCGGC	WF11[a], GLK2[b]
CTACGTCGACGTGCTATGCTAATCGTTCTTCCACACGGATC	GTGCTATGC	WF12[a], GLK3[b]
CTACGTCGACGTGTACTAGCTAATCGTTCTTCCACACGGATC	GTGTACTAGC	WG1[a]
CTACGTCGACTACACTAAGCTAATCGTTCTTCCACACGGATC	TACACTAAGC	WG2[a]
CTACGTCGACTACAGAGAGCTAATCGTTCTTCCACACGGATC	TACAGAGAGC	WG3[a], GAK1[b]
CTACGTCGACTACGACTAGCTAATCGTTCTTCCACACGGATC	TACGACTAGC	WG4[a], GAK2[b]

Sequence	Barcode	Use
CTACGTCGACTAGAGCAAGCTAATCGTTCTTCCACACGGATC	TAGAGCAAGC	WG5[a]
CTACGTCGACTAGCTACAGCTAATCGTTCTTCCACACGGATC	TAGCTACAGC	WG6[a], CAK3[b]
CTACGTCGACTAGTCGTAGCTAATCGTTCTTCCACACGGATC	TAGTCGTAGC	WG7[a]
CTACGTCGACTATATGTAGCTAATCGTTCTTCCACACGGATC	TATATGTAGC	WG8[a]
CTACGTCGACTATCGCGAGCTAATCGTTCTTCCACACGGATC	TATCGCGAGC	WG9[a], MLK1[b]
CTACGTCGACTATGCACAGCTAATCGTTCTTCCACACGGATC	TATGCACAGC	WG10[a], MLK2[b]
CTACGTCGACTCACGATAGCTAATCGTTCTTCCACACGGATC	TCACGATAGC	WG11[a], MLK3[b]
CTACGTCGACTCATAGCAGCTAATCGTTCTTCCACACGGATC	TCATAGCAGC	WG12[a], MAK1
CTACGTCGACTCATGTAAGCTAATCGTTCTTCCACACGGATC	TCATGTAAGC	WH1[a]
CTACGTCGACTCGACATAGCTAATCGTTCTTCCACACGGATC	TCGACATAGC	WH2[a]
CTACGTCGACTCGCACAAGCTAATCGTTCTTCCACACGGATC	TCGCACAAGC	WH3[a]
CTACGTCGACTCTATAGAGCTAATCGTTCTTCCACACGGATC	TCTATAGAGC	WH4[a], MAK2[b]
CTACGTCGACTCTGACGAGCTAATCGTTCTTCCACACGGATC	TCTGACGAGC	WH5[a], MAK3[b]
CTACGTCGACTGAGTAGAGCTAATCGTTCTTCCACACGGATC	TGAGTAGAGC	WH6[a], NLK1[b]
CTACGTCGACTGCATACAGCTAATCGTTCTTCCACACGGATC	TGCATACAGC	WH7[a], NLK2[b]
CTACGTCGACTGCGTCAAGCTAATCGTTCTTCCACACGGATC	TGCGTCAAGC	WH8[a], NLK3[b]
CTACGTCGACTGCTCGAAGCTAATCGTTCTTCCACACGGATC	TGCTCGAAGC	WH9[a], NAK1[b]

Sequence	Barcode	Use
CTACGTCGACTGCTGTGAGCTAATCGTTCTTCCACACGGATC	TGCTGTGAGC	WH10[a], NAK2[b]
CTACGTCGACTGTAGTCAGCTAATCGTTCTTCCACACGGATC	TGTAGTCAGC	WH11[a], NAK3[b]
CTACGTCGACTGTCAGTAGCTAATCGTTCTTCCACACGGATC	TGTCAGTAGC	WH12[a]
ACGTACAATTGACGATGTTGAGAACGGTTCGGCATTG	ACGAT	Cbf1 P1[a]
ACGTACAATTGACGCAGTTGAGAACGGTTCGGCATTG	ACGCA	Cbf1 P2[a]
ACGTACAATTGACGTGGTTGAGAACGGTTCGGCATTG	ACGTG	Cbf1 P3[a]
ACGTACAATTGAGCGCGTTGAGAACGGTTCGGCATTG	AGCGC	Cbf1 P4[a]
ACGTACAATTGAGCTGGTTGAGAACGGTTCGGCATTG	AGCTG	Cbf1 P5[a]
ACGTACAATTGAGTCGGTTGAGAACGGTTCGGCATTG	AGTCG	Cbf1 P6[a]
ACGTACAATTGATATGGTTGAGAACGGTTCGGCATTG	ATATG	Cbf1 P7[a]
ACGTACAATTGATGACGTTGAGAACGGTTCGGCATTG	ATGAC	Cbf1 P8[a]
ACGTACAATTGATGTAGTTGAGAACGGTTCGGCATTG	ATGTA	Cbf1 P9[a]
ACGTACAATTGCACGAGTTGAGAACGGTTCGGCATTG	CACGA	Cbf1 P10[a]
ACGTACAATTGCAGATTGTTGAGAACGGTTCGGCATTG	CAGATT	Met31 P1[a]
ACGTACAATTGCAGTGTGTTGAGAACGGTTCGGCATTG	CAGTGT	Met31 P2[a]
ACGTACAATTGCGACATGTTGAGAACGGTTCGGCATTG	CGACAT	Met31 P3[a]
ACGTACAATTGCGAGCTGTTGAGAACGGTTCGGCATTG	CGAGCT	Met31 P4[a]
ACGTACAATTGCGTAGTGTTGAGAACGGTTCGGCATTG	CGTAGT	Met31 P5[a]
ACGTACAATTGCGTCTTGTTGAGAACGGTTCGGCATTG	CGTCTT	Met31 P6[a]
ACGTACAATTGCGTGATGTTGAGAACGGTTCGGCATTG	CGTGAT	Met31 P7[a]
ACGTACAATTGCTCTATGTTGAGAACGGTTCGGCATTG	CTCTAT	Met31 P8[a]
ACGTACAATTGCTGAGTGTTGAGAACGGTTCGGCATTG	CTGAGT	Met31 P9[a]
ACGTACAATTGCTGTCTGTTGAGAACGGTTCGGCATTG	CTGTCT	Met31 P10[a]

Sequence	Barcode	Use
ACGTACAATTGGACATACGTTGAGAACGGTTCGGCATTG	GACATAC	Nrg1 P1[a]
ACGTACAATTGGACTGACGTTGAGAACGGTTCGGCATTG	GACTGAC	Nrg1 P2[a]
ACGTACAATTGGATCGACGTTGAGAACGGTTCGGCATTG	GATCGAC	Nrg1 P3[a]
ACGTACAATTGGATGTACGTTGAGAACGGTTCGGCATTG	GATGTAC	Nrg1 P4[a]
ACGTACAATTGGCACAACGTTGAGAACGGTTCGGCATTG	GCACAAC	Nrg1 P5[a]
ACGTACAATTGGCGACACGTTGAGAACGGTTCGGCATTG	GCGACAC	Nrg1 P6[a]
ACGTACAATTGGCGCGACGTTGAGAACGGTTCGGCATTG	GCGCGAC	Nrg1 P7[a]
ACGTACAATTGGCGTAACGTTGAGAACGGTTCGGCATTG	GCGTAAC	Nrg1 P8[a]
ACGTACAATTGGTACGACGTTGAGAACGGTTCGGCATTG	GTACGAC	Nrg1 P9[a]
ACGTACAATTGGTGATACGTTGAGAACGGTTCGGCATTG	GTGATAC	Nrg1 P10[a]
ACGTACAATTGGTGTGCACGTTGAGAACGGTTCGGCATTG	GTGTGCAC	Gcn4 P1[a]
ACGTACAATTGTAGCGCACGTTGAGAACGGTTCGGCATTG	TAGCGCAC	Gcn4 P2[a]
ACGTACAATTGTATAGCACGTTGAGAACGGTTCGGCATTG	TATAGCAC	Gcn4 P3[a]
ACGTACAATTGTATCTCACGTTGAGAACGGTTCGGCATTG	TATCTCAC	Gcn4 P4[a]
ACGTACAATTGTATGACACGTTGAGAACGGTTCGGCATTG	TATGACAC	Gcn4 P5[a]
ACGTACAATTGTCGAGCACGTTGAGAACGGTTCGGCATTG	TCGAGCAC	Gcn4 P6[a]
ACGTACAATTGTCTATCACGTTGAGAACGGTTCGGCATTG	TCTATCAC	Gcn4 P7[a]
ACGTACAATTGTCTGCCACGTTGAGAACGGTTCGGCATTG	TCTGCCAC	Gcn4 P8[a]
ACGTACAATTGTGACGCACGTTGAGAACGGTTCGGCATTG	TGACGCAC	Gcn4 P9[a]
ACGTACAATTGTGAGTCACGTTGAGAACGGTTCGGCATTG	TGAGTCAC	Gcn4 P10[a]

[a]W = Well and P = Plate, so WA1 is Well A1 and P1 is Plate 1.

[b]ChIPed sample barcode: C=Cbf1-tagged; G=Gcn4-tagged; A=AAS; L=Glucose; K=Input; P=IP; 1-3=Sample replicate. So CLP1=Cbf1-tagged IP in glucose, replicate 1.

Synthetic promoters were amplified by using one well-specific PCR primer and one plate-specific PCR primer. Custom adapters were ligated on to the products and sequenced on an Illumina MiSEQ machine.

A subset of well-specific primers were reused to barcode CHiP samples for multiplexing on an Illumina HiSEQ 2000. All primers are listed in 5’-3’ order.

Table S2: Oligonucleotides used for strain manipulation, validation, PCR, and sequencing

Name	Sequence	Purpose
RZ84	5’-GATCGTATCACGTGCTTTAC-3’	Cbf1 site, forward
RZ85	3’-CATAGTGCACGAAATGCTAG-5’	Cbf1 site, reverse
RZ86	5’-GATCGTAATGACTCATTTAC-3’	Gcn4 site, forward
RZ87	3’-CATTACTGAGTAAATGCTAG-5’	Gcn4 site, reverse
RZ88	5’-GATCGTAGCCACAGTTTTAC-3’	Met 31/32 site, forward
RZ89	3’-CATCGGTGTCAAAATGCTAG-5’	Met 31/32 site, reverse
RZ90	5’-GATCGTATGAGGACCCTTAC-3’	Nrg1 site, forward
RZ91	3’-CATACTCCTGGGAATGCTAG-5’	Nrg1 site, reverse
RZ92	5’-CATTCTTACCCACTCCTGTTCTAG -3’	Gcn4 Avi-tagging check, upstream PCR primer
RZ93	5’-CGCGTCTGACTTCTAATCAGAAG-3’	Gcn4 Avi-tagging check, downstream PCR primer
RZ94	5’-CCGATGAAGCAAACATCGAAAAG -3’	Cbf1 Avi-tagging check, upstream PCR primer
RZ95	5’-TCCGTCCCGTCCTCTTTTAC -3’	Cbf1 Avi-tagging check, downstream PCR primer
RZ96	5-’CCGGAAAATATGGCTAGAGGTC -3’	Met31 Avi-tagging check, upstream PCR primer
RZ97	5’-GTACGTCACCACTTTGTGCG -3’	Met31 Avi-tagging check, downstream PCR primer

Name	Sequence	Purpose
RZ98	5'-CGGAAGCAAAGAACAGATCCA -3'	Nrg1 Avi-tagging check, upstream PCR primer
RZ99	5'-CCAGACATGATCTTAAGCGGAAG -3'	Nrg1 Avi-tagging check, downstream PCR primer
RZ127	5'-GACATGATAATTGCTTGCAACACTATAGAACACATTTGAAAAAGGGACAAGGGATCGAGCAGAAGCTGAT -3'	myc-C-Avi tagging primer, Nrg1, upstream
RZ128	5'-AGTGCGGAATAGTAGTACTGCTAATGAGAAAAACACGGGTATACCGTCAACTGCAGGTCGACAACCCTTAAT -3'	myc-C-Avi tagging primer, Nrg1, downstream
RZ129	5'-AACAAGAGAACGAAAGAAAAAGCACTAGGAGCGATAATCCACATGAGGCTGGGATCGAGCAGAAGCTGAT -3'	myc-C-Avi tagging primer, Cbf1, upstream
RZ130	5'-GTGCTATGGGGCAGAGACGCAGATACATAGGGAGACTCGAAATACATTTACTGCAGGTCGACAACCCTTAAT -3'	myc-C-Avi tagging primer, Cbf1, downstream
RZ131	5'-CTTTTTTGTGCCTTTGTTACGTCTATATTCTATTGAAACTGGAGCTCGTTTTCGACACTGG -3'	Insert PCORE into lys-2, upstream PCR primer
RZ132	5'-TATTATATATTATTCTCGGAGTTTTTAAGTGACATCACCCTCCTTACCATTAAGTTGATC -3'	Insert PCORE into lys-2, downstream PCR primer
RZ133	5'-CTTTTTTGTGCCTTTCTTACGTCTATATTCATTGAAACTGGACTGGGTCATGGCTGCG -3'	Insert BirA into lys-2, upstream PCR primer
RZ134	5'-TATTATATATTATTCTCGGAGTTTTTAAGTGACATCACCCAAGCTTGCAAATTAAAGCCTTCGAG -3'	Insert BirA into lys-2, downstream PCR primer

Name	Sequence	Purpose
RZ135	5’-GCTCATCAAGGATGCGATAAAGAATGGTACCGGCCTGTTGGGGATCGAGCAGAAGCTGAT -3’	myc-C-Avi tagging primer, Met31, upstream
RZ136	5’-ATTCTACTTATCTCAATGGCTAAAGTATATATCTATCTATCTGCAGGTCGACAACCCTTAAT -3’	myc-C-Avi tagging primer, Met31, downstream
RZ137	5’-AAATGAGGTTGCCAGATTAAAGAAATTAGTTGGCGAACGCGGGATCGAGCAGAAGCTGAT -3’	myc-C-Avi tagging primer, Gcn4, upstream
RZ138	5’-GCGTGGTGTAAAATTCTACTTAAGAAAATTGGCATAAAAACTGCAGGTCGACAACCCTTAAT -3’	myc-C-Avi tagging primer, Gcn4, downstream
RZ143	5’-CGACCTCATGCTATACCTGAGAAAG -3’	myc-C-avi integration check PCR primer, upstream, internal to tag
RZ144	5’-TGGGGATGTATGGGCTAAATGTAC -3’	myc-C-Avi integration check PCR primer, downstream, internal to Kan
RZ147	5’-GCAGTTGCTTTCTCCTATGGGAAG -3’	PCORE and BirA integration check PCR primer, upstream
RZ148	5’-GAATTGGTCAGTATCGACCTGTGAA -3’	PCORE and Bira integration check PCR primer, downstream
RZ149	5’-GTTAGAAGAAAAGAGTCGGGATCTCTG -3’	BirA integration check PCR primer, upstream, internal to BirA

Name	Sequence	Purpose
RZ150	5'-CTGTACAGACGCGTGTACGC -3'	BirA integration check PCR primer, downstream, internal to BirA
RZ151	5'-TTAAGTCCGGGGATCCCCAG -3'	Universal myc-C-Avi-tag sequencing primer, internal to Avi tag.
RZ158	5'-GGGAGGAGTCATGGCAAATA -3'	Cbf1 ChIP check qPCR primer: ADE765.
RZ159	5'-CGTATACGGTGACGACGAGA -3'	5' PRIMER AROUND ADE756 SET 2 SET 4
RZ169	5'-TAGGGGCTTAGCATCCACAC -3'	SUC2 qPCR Primer
RZ170	5'-TGGATACCTTCGACAGCTCA -3'	SUC2 qPCR Primer
RZ177	5'-CCCCTAAACATTCAGATTGTAAAC -3'	Gcn4 ChIP check qPCR primer (YJR109C)
RZ178	5'-TCTCGATGCTTACTCAAGGTG -3'	Gcn4 ChiP check qPCR primer (YJR109C)
RZ183	5'-GCCGCCACAGAAAACTTAC -3'	Met31 ChIP check qPCR primer (YNL278W)
RZ184	5'-GAGCTATGGGCAATTGTACG -3'	Met31 ChiP check qPCR primer (YNL278W)
RZ193	5'-CCGGAAAAGAAGGGAAAAAT -3'	Nrg1 ChIP check qPCR primer (YDR043C)

Name	Sequence	Purpose
RZ194	5’-CCTGCAGCCAGACTGTAGAA -3’	Nrg1 ChIP check qPCR primer (YDR043C)
RZ226	5’-CCCTCGTTCAATTGCTCACCTCGAC -3’	Custom read 1 sequencing primer for sequencing synthetic promoters.
RZ227	5’-GCTCCCCATTTCACGAATTG-3’	Custom read 2 sequencing primer for synthetic promoters
RZ230	/5Phos/ TCGAGGTGAGCAATTGAACGAGGGGTGTAGATCTC GGTGGTCGCCGTATCATT -3’	Read 1 flow cell adapter and sequencing primer
RZ231	/5Phos/ AATTCGTGAAATGGGGAGCATCTCGTATGCCGTCTT CTGCTTG -3’	Read 2 flow cell adapter and sequencing primer
RZ232	5’- AATGATACGGCGACCACCGAGATCTACACCCCTCGT TCAATTGCTCACC -3’	Read 1 flow cell adapter and sequencing primer (reverse complement)
RZ233	5’- CAAGCAGAAGACGGCATACGAGATGCTCCCCATTTC ACG -3’	Read 2 flow cell adapter and sequencing primer (reverse complement)
RZ257.1	5’- CAAGCAGAAGACGGCATACGAGATCGGTCTCGGCA TTCCTGCTGAACCGCTCTTCCGATCTGTTGAGAACG GTTCGGCATTG -3’	Downstream pcr primer for synthetic promoter amplification for sequencing post-ChIP (1/4)

Name	Sequence	Purpose
RZ257.2	5'-CAAGCAGAAGACGGCATACGACGATCGGTCTCGGCATTCCTGCTGAACCGCTCTTCCGATCTCGTTGAGAACGGTTCGGCATTG -3'	Downstream pcr primer for synthetic promoter amplification for sequencing post-ChIP (2/4)
RZ257.3	5'-CAAGCAGAAGACGGCATACGAGATCGGTCTCGGCATTCCTGCTGAACCGCTCTTCCGATCTTAGTTGAGAACGGTTCGGCATTG -3'	Downstream pcr primer for synthetic promoter amplification for sequencing post-ChIP (3/4)
RZ257.4	5'-CAAGCAGAAGACGGCATACGAGATCGGTCTCGGCATTCCTGCTGAACCGCTCTTCCGATCTACAGTTGAGAACGGTTCGGCATTG -3'	Downstream pcr primer for synthetic promoter amplification for sequencing post-ChIP (4/4)
RZ259	5'- TGTAATCGTTCTTCCACACGGATC -3'	qPCR Primer for library concentration check, post prep.
RZ260	5'- TTCCTGCTGAACCGCTCTTC-3'	qPCR Primer for library concentration check, post prep.

Table S3: List of all promoters and condition-specific expression and occupancy values

Promoter	Glucose Expression	Glucose Occupancy	AAS Expression	AAS Occupancy	Tagged Factor
c	4.48	1.96	4.48	1.92	Cbf1
C	4.87	2.15	4.51	2.11	Cbf1
cC	6.50	4.71	NA	4.27	Cbf1
cCcNC	6.84	8.60	NA	7.40	Cbf1
cCnGGn	3.34	3.04	5.89	4.04	Cbf1
cCNm	6.19	4.07	NA	4.09	Cbf1
cg	2.82	1.69	3.16	1.96	Cbf1
Cg	6.28	1.85	NA	2.14	Cbf1
cgg	2.06	0.03	5.04	0.03	Cbf1
cgGm	3.21	1.28	NA	1.63	Cbf1
CGGn	2.03	1.34	NA	1.70	Cbf1
cgm	6.62	1.57	NA	1.87	Cbf1
CgM	3.77	0.05	NA	0.05	Cbf1
cGN	3.96	1.30	NA	1.65	Cbf1
CgnM	4.04	NA	NA	NA	Cbf1
cGnmM	5.47	1.58	NA	2.14	Cbf1
cgnN	1.80	0.85	4.34	1.38	Cbf1
cm	4.13	3.50	NA	2.68	Cbf1
CMcC	2.70	0.05	5.18	0.05	Cbf1
cmG	6.48	1.85	NA	2.18	Cbf1
cMgN	1.00	0.04	NA	0.05	Cbf1
cmM	6.05	2.28	NA	2.55	Cbf1

Promoter	Glucose Expression	Glucose Occupancy	AAS Expression	AAS Occupancy	Tagged Factor
cmn	3.77	1.64	5.20	2.01	Cbf1
CMn	4.40	2.00	5.62	2.38	Cbf1
CMNgC	2.22	0.05	NA	0.05	Cbf1
CMnmg	5.88	1.78	NA	2.31	Cbf1
CMNMn	3.80	1.84	4.51	2.30	Cbf1
cN	3.94	1.66	4.20	1.91	Cbf1
Cn	2.91	1.97	3.13	2.08	Cbf1
CN	3.96	1.89	4.17	2.09	Cbf1
cNCCM	4.28	0.04	NA	0.04	Cbf1
Cng	4.68	1.48	NA	1.83	Cbf1
cngCnG	4.77	3.52	NA	3.63	Cbf1
cnGN	1.66	0.70	3.49	1.17	Cbf1
CNGNmn	0.89	0.72	1.77	1.01	Cbf1
cnm	6.83	NA	NA	NA	Cbf1
cNM	4.00	1.68	NA	2.09	Cbf1
cnNm	2.45	1.06	3.54	1.34	Cbf1
g	1.73	0.10	3.16	0.10	Cbf1
G	1.60	0.10	3.06	0.09	Cbf1
gc	3.00	0.05	6.67	0.05	Cbf1
Gc	6.25	2.08	NA	2.26	Cbf1
GcgG	3.60	1.58	NA	1.61	Cbf1
GCgggn	2.41	1.78	NA	1.52	Cbf1
gcmm	7.61	1.96	NA	2.30	Cbf1

Promoter	Glucose Expression	Glucose Occupancy	AAS Expression	AAS Occupancy	Tagged Factor
gCN	4.57	1.76	6.52	1.98	Cbf1
Gcn	4.41	1.61	NA	1.82	Cbf1
gg	2.80	0.99	NA	0.79	Cbf1
Gg	2.72	0.29	3.88	0.66	Cbf1
GG	2.23	0.10	4.88	0.10	Cbf1
gGCm	4.91	NA	NA	NA	Cbf1
gGCn	5.02	1.89	8.19	2.27	Cbf1
gGm	2.50	0.08	4.75	0.09	Cbf1
gGM	2.04	0.05	NA	0.05	Cbf1
ggMMM	5.15	0.14	NA	0.15	Cbf1
GGmn	1.57	0.09	2.55	0.08	Cbf1
ggNm	1.17	0.04	3.82	0.03	Cbf1
gGnm	1.12	0.10	2.03	0.10	Cbf1
GGNmc	5.27	2.59	NA	3.10	Cbf1
ggnN	0.72	0.07	1.80	0.08	Cbf1
gM	4.55	0.07	NA	0.07	Cbf1
Gm	2.03	0.09	3.39	0.08	Cbf1
GM	2.42	0.45	4.19	0.23	Cbf1
GMC	8.63	2.14	NA	2.35	Cbf1
gMG	2.32	0.33	5.23	0.86	Cbf1
GmGg	2.57	NA	4.88	NA	Cbf1
GmGggN	2.74	NA	4.95	NA	Cbf1
Gmgm	1.95	NA	NA	NA	Cbf1

Promoter	Glucose Expression	Glucose Occupancy	AAS Expression	AAS Occupancy	Tagged Factor
GmgmGG	2.33	NA	NA	NA	Cbf1
gMgn	1.66	0.08	3.32	0.09	Cbf1
gMGn	1.59	0.10	3.20	0.13	Cbf1
Gmgnm	1.39	0.13	2.80	0.08	Cbf1
gmMc	5.72	NA	NA	NA	Cbf1
GmmgC	7.54	3.92	NA	4.15	Cbf1
gn	3.37	1.15	NA	1.65	Cbf1
GN	1.26	0.08	2.47	0.09	Cbf1
gNC	6.22	1.90	NA	2.04	Cbf1
gng	2.04	0.07	3.04	0.09	Cbf1
gNGGNn	1.04	0.10	1.96	0.07	Cbf1
gNM	1.59	0.08	3.98	0.06	Cbf1
GNm	1.20	0.08	2.34	0.08	Cbf1
GNM	1.74	0.09	2.72	0.08	Cbf1
gnmG	6.21	NA	NA	NA	Cbf1
GNmN	3.98	NA	NA	NA	Cbf1
GnnCn	2.79	1.57	3.42	1.87	Cbf1
GnnMCn	4.18	1.95	NA	2.50	Cbf1
m	1.04	0.09	1.30	0.06	Cbf1
M	1.25	0.13	1.42	0.12	Cbf1
mc	5.38	2.28	NA	2.61	Cbf1
mC	6.72	2.27	3.85	2.52	Cbf1
MCG	5.94	2.04	NA	2.32	Cbf1

Promoter	Glucose Expression	Glucose Occupancy	AAS Expression	AAS Occupancy	Tagged Factor
Mcggg	3.90	1.43	NA	1.82	Cbf1
mcm	8.11	2.08	NA	2.31	Cbf1
mCm	5.66	2.36	NA	2.64	Cbf1
MCMgMg	5.41	1.93	NA	2.18	Cbf1
mcn	4.14	1.83	NA	2.32	Cbf1
MCN	5.11	1.88	NA	2.12	Cbf1
MCnCn	1.91	0.04	2.59	0.04	Cbf1
MCnn	3.12	1.68	4.10	2.13	Cbf1
MCNN	1.18	0.05	1.92	0.03	Cbf1
mG	6.85	1.96	NA	2.14	Cbf1
Mg	2.25	0.27	3.86	0.13	Cbf1
MG	2.14	0.16	3.89	0.13	Cbf1
mgc	6.52	2.22	NA	2.44	Cbf1
Mgc	5.96	2.30	NA	2.42	Cbf1
MGCG	4.58	3.22	NA	3.27	Cbf1
MgcM	4.09	0.15	NA	0.65	Cbf1
MGCn	5.74	1.69	NA	2.04	Cbf1
MgG	2.13	0.13	NA	0.10	Cbf1
MGg	2.23	0.10	4.27	0.11	Cbf1
MggCNM	4.83	NA	NA	NA	Cbf1
mggN	1.43	0.09	2.81	0.09	Cbf1
MGGNm	2.14	NA	NA	NA	Cbf1
MGm	3.15	NA	5.13	NA	Cbf1

Promoter	Glucose Expression	Glucose Occupancy	AAS Expression	AAS Occupancy	Tagged Factor
mGmC	3.54	0.06	NA	0.05	Cbf1
MGmCc	2.86	0.08	NA	0.08	Cbf1
MGmM	5.39	0.13	NA	0.11	Cbf1
mgMNc	2.94	0.06	NA	0.01	Cbf1
mGn	0.88	0.04	3.41	0.03	Cbf1
mGNc	5.60	2.20	NA	2.37	Cbf1
MGnGC	5.61	1.99	NA	2.27	Cbf1
mGNNG	1.17	0.09	2.90	0.09	Cbf1
mm	3.70	0.09	4.09	0.08	Cbf1
mM	3.34	0.20	4.94	NA	Cbf1
MM	3.65	0.10	4.51	0.09	Cbf1
MMgc	4.43	0.06	NA	0.04	Cbf1
MMGN	1.56	0.09	3.79	0.10	Cbf1
mmm	4.32	0.10	NA	0.10	Cbf1
mmn	7.73	2.35	NA	2.70	Cbf1
MmnCMN	7.32	2.06	NA	2.05	Cbf1
MmNm	2.78	0.06	4.63	NA	Cbf1
mn	0.63	0.08	0.87	0.07	Cbf1
Mn	0.76	0.09	0.87	0.07	Cbf1
MN	0.95	0.30	1.25	0.21	Cbf1
MNcg	3.92	4.41	NA	5.25	Cbf1
mnCn	1.47	0.03	2.83	0.04	Cbf1
MnG	0.80	0.06	1.33	0.08	Cbf1

Promoter	Glucose Expression	Glucose Occupancy	AAS Expression	AAS Occupancy	Tagged Factor
MngG	1.20	0.04	5.00	0.03	Cbf1
mNGmm	3.31	0.09	NA	0.11	Cbf1
MNm	1.60	NA	2.22	NA	Cbf1
MnMG	1.97	0.49	4.33	0.19	Cbf1
MNMG	1.32	0.12	NA	0.10	Cbf1
mnMGG	2.20	0.10	4.10	0.09	Cbf1
mNnc	1.48	0.04	2.18	0.03	Cbf1
MNnc	4.00	2.11	4.55	2.34	Cbf1
mnng	0.79	0.07	1.36	0.07	Cbf1
MnNgnG	1.02	0.08	1.61	0.09	Cbf1
n	0.61	0.11	0.59	0.06	Cbf1
N	0.84	0.10	0.89	0.07	Cbf1
nc	4.39	2.01	3.98	2.22	Cbf1
nC	4.63	1.90	3.96	2.14	Cbf1
Nc	2.36	0.19	NA	0.31	Cbf1
NC	1.96	0.47	2.13	0.63	Cbf1
NcG	5.31	1.81	NA	2.08	Cbf1
NCg	5.77	1.75	NA	1.92	Cbf1
NcGnGN	1.43	0.69	4.07	1.11	Cbf1
ncGNNm	1.36	0.72	3.43	0.99	Cbf1
ncM	4.80	1.88	NA	2.30	Cbf1
Ncm	5.31	1.81	7.46	2.17	Cbf1
NCmn	1.53	NA	4.29	NA	Cbf1

Promoter	Glucose Expression	Glucose Occupancy	AAS Expression	AAS Occupancy	Tagged Factor
nCmnGm	5.59	1.14	NA	2.16	Cbf1
ncN	3.29	5.89	3.67	9.60	Cbf1
NCngG	2.19	1.13	4.98	1.51	Cbf1
nCnGm	3.22	1.08	6.55	1.77	Cbf1
nCNGNG	0.86	0.03	4.05	0.04	Cbf1
nCnm	3.98	1.42	5.49	1.87	Cbf1
nCNMCn	6.45	3.89	NA	4.19	Cbf1
ng	1.12	0.08	2.79	0.07	Cbf1
nG	1.14	0.08	2.66	0.07	Cbf1
Ng	1.52	0.11	3.01	0.11	Cbf1
NG	1.21	0.16	2.63	0.21	Cbf1
ngc	5.78	2.02	8.40	2.15	Cbf1
NGc	5.42	2.18	NA	2.50	Cbf1
NgCgg	5.14	NA	NA	NA	Cbf1
nGcM	2.89	0.05	NA	0.05	Cbf1
nGcNm	4.86	1.20	NA	2.00	Cbf1
ngg	1.34	0.05	4.43	0.03	Cbf1
Ngg	1.52	0.16	2.64	0.18	Cbf1
nGgCNn	1.13	0.07	3.39	0.03	Cbf1
ngGm	6.60	0.09	NA	0.07	Cbf1
ngM	1.56	0.09	3.43	0.08	Cbf1
nGm	1.38	0.08	2.83	0.09	Cbf1
nGMGMC	7.00	2.46	NA	2.88	Cbf1

Promoter	Glucose Expression	Glucose Occupancy	AAS Expression	AAS Occupancy	Tagged Factor
NGn	0.69	NA	1.30	NA	Cbf1
NGnc	7.09	NA	NA	NA	Cbf1
NgNGG	1.26	0.08	4.15	0.08	Cbf1
nm	0.83	0.11	1.10	0.11	Cbf1
Nm	0.96	0.08	1.20	0.08	Cbf1
NM	1.51	0.14	NA	0.12	Cbf1
nMC	6.31	2.15	8.06	2.36	Cbf1
nMcm	6.16	2.25	NA	2.71	Cbf1
nmg	1.37	0.09	2.44	0.11	Cbf1
nMG	1.23	0.03	5.17	0.03	Cbf1
nMgm	1.83	0.09	2.99	0.09	Cbf1
nmGN	0.92	0.16	1.46	0.50	Cbf1
nmM	4.04	0.06	NA	0.04	Cbf1
nMm	2.57	0.09	3.54	0.09	Cbf1
NMM	5.12	1.62	NA	1.70	Cbf1
NMMgn	4.82	NA	NA	NA	Cbf1
nmn	0.49	0.07	0.58	0.07	Cbf1
Nmn	8.41	NA	NA	NA	Cbf1
nmnM	1.38	0.11	2.08	0.06	Cbf1
nN	0.63	0.42	0.69	0.38	Cbf1
NN	0.70	0.08	0.98	0.07	Cbf1
nnC	3.98	1.95	4.41	2.14	Cbf1
nNC	9.86	2.60	6.35	2.80	Cbf1

Promoter	Glucose Expression	Glucose Occupancy	AAS Expression	AAS Occupancy	Tagged Factor
NNc	4.12	2.11	3.88	2.47	Cbf1
NNC	4.29	2.03	3.64	2.15	Cbf1
NNcc	6.53	4.81	NA	4.59	Cbf1
NncgN	3.22	0.98	5.88	1.51	Cbf1
NNCN	3.42	1.71	3.25	2.01	Cbf1
NNG	1.21	0.13	2.46	0.10	Cbf1
Nngc	4.16	2.13	NA	2.40	Cbf1
nnGGg	1.61	0.13	4.91	0.15	Cbf1
NnM	0.86	0.08	1.08	0.06	Cbf1
NNm	0.71	0.07	0.86	0.07	Cbf1
NNn	0.38	NA	0.39	0.31	Cbf1
Basal	NA	0.17	0.98	0.07	Cbf1
cc	NA	4.50	NA	4.33	Cbf1
CC	NA	4.89	NA	4.51	Cbf1
Cccgcn	NA	8.11	NA	7.22	Cbf1
CccMM	NA	6.04	NA	5.92	Cbf1
ccm	NA	4.28	NA	4.41	Cbf1
ccNgc	NA	6.63	NA	6.51	Cbf1
ccnGM	NA	3.85	1.38	3.63	Cbf1
cGcGmm	NA	4.65	NA	4.56	Cbf1
cgcM	NA	4.40	NA	4.21	Cbf1
cGmCCg	NA	6.25	NA	5.44	Cbf1
cM	NA	5.70	NA	6.34	Cbf1

Promoter	Glucose Expression	Glucose Occupancy	AAS Expression	AAS Occupancy	Tagged Factor
CMCGCM	NA	7.28	NA	8.04	Cbf1
CMCm	NA	4.79	NA	4.82	Cbf1
CMCn	NA	4.47	NA	4.52	Cbf1
cMgM	NA	2.10	NA	2.47	Cbf1
CMMm	NA	0.09	NA	0.05	Cbf1
cmNc	NA	4.51	NA	4.80	Cbf1
cnCC	NA	7.20	NA	9.34	Cbf1
cncg	NA	3.92	NA	4.17	Cbf1
cNcG	NA	4.17	NA	4.32	Cbf1
CNCN	NA	4.34	NA	4.39	Cbf1
CNm	NA	2.19	NA	2.20	Cbf1
GC	NA	5.65	NA	5.64	Cbf1
GcC	NA	4.38	NA	4.51	Cbf1
gcgcN	NA	4.11	NA	4.10	Cbf1
gCGGN	NA	0.94	NA	1.30	Cbf1
gcgmGn	NA	1.16	NA	1.62	Cbf1
GCM	NA	7.04	NA	6.61	Cbf1
GcMNc	NA	5.01	NA	4.85	Cbf1
gCnGc	NA	4.41	NA	4.55	Cbf1
gCNnn	NA	0.93	NA	1.42	Cbf1
ggCMc	NA	4.91	NA	4.62	Cbf1
gGMGN	NA	1.93	NA	3.63	Cbf1
gGnccm	NA	4.67	NA	4.29	Cbf1

Promoter	Glucose Expression	Glucose Occupancy	AAS Expression	AAS Occupancy	Tagged Factor
GMCn	NA	2.17	NA	1.95	Cbf1
GmGcCN	NA	4.47	NA	4.64	Cbf1
gmN	NA	0.95	NA	2.07	Cbf1
GmN	NA	2.01	NA	2.03	Cbf1
GNCC	NA	6.89	NA	6.77	Cbf1
GNNc	NA	2.14	NA	2.23	Cbf1
mcC	NA	4.83	NA	4.94	Cbf1
MCcG	NA	3.39	NA	3.26	Cbf1
MCCG	NA	4.56	NA	4.08	Cbf1
MccN	NA	4.52	NA	4.34	Cbf1
McG	NA	1.87	NA	2.34	Cbf1
mCmC	NA	5.48	NA	5.53	Cbf1
MCMC	NA	4.74	NA	4.58	Cbf1
MCMccg	NA	6.75	NA	6.18	Cbf1
MCMMNc	NA	5.30	NA	5.23	Cbf1
MGc	NA	7.50	NA	7.33	Cbf1
MGCCcN	NA	6.50	NA	5.65	Cbf1
MGccnc	NA	6.80	NA	6.32	Cbf1
mgcmC	NA	5.07	NA	5.06	Cbf1
mGCN	NA	7.95	NA	7.21	Cbf1
mGggC	NA	2.09	NA	2.36	Cbf1
mGMn	NA	0.07	NA	0.11	Cbf1
MgN	NA	8.37	NA	5.80	Cbf1

Promoter	Glucose Expression	Glucose Occupancy	AAS Expression	AAS Occupancy	Tagged Factor
MGnnNM	NA	0.09	NA	0.08	Cbf1
Mm	NA	0.46	NA	NA	Cbf1
MMgC	NA	0.07	NA	0.06	Cbf1
mmnmC	NA	2.58	NA	2.77	Cbf1
MnccMM	NA	4.79	NA	4.55	Cbf1
MNgMmn	NA	0.08	NA	0.07	Cbf1
mNm	NA	0.17	NA	0.16	Cbf1
mnmC	NA	2.76	NA	2.95	Cbf1
MNmmCN	NA	2.52	NA	2.44	Cbf1
ncc	NA	4.47	NA	4.43	Cbf1
nCCGnm	NA	4.15	NA	3.93	Cbf1
NCcmg	NA	4.26	NA	4.28	Cbf1
ncg	NA	1.38	NA	1.68	Cbf1
NCgGg	NA	1.55	NA	1.76	Cbf1
ncGgm	NA	1.10	NA	1.61	Cbf1
ncGmm	NA	1.83	NA	2.07	Cbf1
ngC	NA	2.56	NA	2.85	Cbf1
ngCc	NA	4.75	NA	4.60	Cbf1
Ngm	NA	0.06	NA	0.09	Cbf1
nGMC	NA	7.20	NA	8.63	Cbf1
NgNm	NA	3.94	NA	5.05	Cbf1
NMcc	NA	4.73	NA	4.83	Cbf1
nmCGC	NA	10.81	NA	10.28	Cbf1

Promoter	Glucose Expression	Glucose Occupancy	AAS Expression	AAS Occupancy	Tagged Factor
Nmg	NA	4.27	NA	5.18	Cbf1
nmgG	NA	0.20	NA	0.14	Cbf1
NmmgMC	NA	2.64	NA	2.88	Cbf1
nMNMm	NA	0.10	NA	0.13	Cbf1
nn	NA	0.29	NA	0.10	Cbf1
Nn	NA	0.27	NA	NA	Cbf1
NNgCgM	NA	4.56	NA	4.41	Cbf1
NNGn	NA	0.10	NA	0.07	Cbf1
cCCC	NA	NA	NA	11.18	Cbf1
mGCM	NA	NA	NA	4.44	Cbf1
NccmN	NA	NA	NA	4.73	Cbf1
NGccmN	NA	NA	NA	6.80	Cbf1
c	2.18	0.86	1.94	0.15	Gcn4
C	2.15	0.85	1.78	0.20	Gcn4
cc	2.67	0.69	2.21	0.19	Gcn4
CC	2.30	1.05	2.19	0.19	Gcn4
Ccc	3.02	NA	2.01	NA	Gcn4
Cccgcn	2.72	NA	4.67	1.80	Gcn4
cCcNC	1.13	NA	NA	NA	Gcn4
CcG	3.07	NA	NA	3.11	Gcn4
CCG	2.20	NA	NA	10.36	Gcn4
ccgC	2.37	NA	NA	NA	Gcn4
Ccm	3.10	0.73	4.40	0.17	Gcn4

Promoter	Glucose Expression	Glucose Occupancy	AAS Expression	AAS Occupancy	Tagged Factor
ccN	2.59	0.65	2.47	0.07	Gcn4
cCN	2.45	0.91	1.69	0.14	Gcn4
cCnGGn	0.59	NA	NA	4.11	Gcn4
cCNm	2.83	0.78	5.00	0.11	Gcn4
cg	1.05	NA	NA	NA	Gcn4
Cg	2.52	1.40	NA	2.27	Gcn4
Cgcm	2.78	1.80	NA	2.16	Gcn4
cGmCCg	2.47	2.44	NA	6.14	Gcn4
cGN	1.30	1.02	5.05	1.48	Gcn4
cGnmM	4.28	1.21	NA	2.29	Gcn4
cm	2.79	0.63	3.99	0.16	Gcn4
cM	0.79	3.20	5.60	6.27	Gcn4
CM	3.16	0.75	NA	0.39	Gcn4
CMCGCM	2.74	2.15	NA	3.13	Gcn4
CMCm	4.48	0.54	NA	0.13	Gcn4
CMCn	2.47	0.64	3.92	0.11	Gcn4
cmG	2.25	1.27	NA	2.39	Gcn4
cmM	5.79	0.70	NA	0.16	Gcn4
cmn	1.69	0.57	3.45	0.13	Gcn4
cmNc	2.39	0.71	NA	0.16	Gcn4
cmnmc	3.37	0.42	5.17	0.10	Gcn4
CMNn	3.58	NA	NA	NA	Gcn4
cn	1.12	0.66	NA	0.08	Gcn4

Promoter	Glucose Expression	Glucose Occupancy	AAS Expression	AAS Occupancy	Tagged Factor
cN	2.45	0.67	3.03	0.13	Gcn4
CN	1.89	0.87	1.78	0.12	Gcn4
CnC	5.70	NA	NA	NA	Gcn4
cNcG	2.90	2.43	NA	2.21	Gcn4
Cncgm	2.65	NA	NA	1.94	Gcn4
cng	1.38	0.88	5.13	1.19	Gcn4
Cng	1.26	1.02	NA	1.62	Gcn4
CnG	1.26	1.04	5.21	1.43	Gcn4
cngCnG	1.22	0.85	NA	4.29	Gcn4
cnMc	3.12	0.43	3.40	0.12	Gcn4
CNMgg	1.05	1.08	NA	5.48	Gcn4
cnncMN	2.19	0.55	4.30	0.10	Gcn4
g	1.15	1.10	4.02	1.49	Gcn4
G	1.18	1.24	3.79	1.67	Gcn4
Gc	2.15	1.21	4.57	2.15	Gcn4
GC	2.74	2.64	NA	9.48	Gcn4
GcC	2.56	1.32	4.32	2.01	Gcn4
gcgCM	2.75	1.73	NA	6.28	Gcn4
gcgcN	1.97	1.16	NA	6.67	Gcn4
GcgG	1.25	4.48	NA	14.26	Gcn4
GCgggn	1.03	NA	NA	14.77	Gcn4
gCM	3.38	0.79	NA	2.15	Gcn4
Gcm	2.79	0.95	NA	2.14	Gcn4

Promoter	Glucose Expression	Glucose Occupancy	AAS Expression	AAS Occupancy	Tagged Factor
GCm	2.49	0.75	NA	2.15	Gcn4
GCM	3.07	NA	NA	NA	Gcn4
Gcn	1.62	0.89	3.98	1.42	Gcn4
gCnGc	2.59	1.53	NA	5.09	Gcn4
gCNnn	0.37	0.70	1.29	0.50	Gcn4
Gg	0.93	NA	5.05	10.26	Gcn4
GG	0.89	1.35	5.43	5.37	Gcn4
gGCn	1.12	3.13	NA	7.13	Gcn4
GggnG	0.71	1.17	NA	11.69	Gcn4
gGm	1.68	2.32	NA	4.54	Gcn4
gGMGN	1.86	NA	NA	14.85	Gcn4
ggMMM	5.08	1.98	NA	10.28	Gcn4
GGmn	0.52	0.95	4.41	3.12	Gcn4
gM	1.71	0.88	5.07	1.29	Gcn4
Gm	1.70	1.17	5.53	1.12	Gcn4
GM	1.58	1.27	NA	1.49	Gcn4
gmc	2.81	1.06	NA	2.72	Gcn4
gmC	2.64	0.78	NA	2.47	Gcn4
GMC	2.62	1.13	NA	2.49	Gcn4
gMG	1.60	NA	NA	NA	Gcn4
GmGg	1.73	1.61	NA	7.10	Gcn4
Gmgm	1.94	NA	NA	NA	Gcn4
GmgmGg	1.20	NA	NA	NA	Gcn4

Promoter	Glucose Expression	Glucose Occupancy	AAS Expression	AAS Occupancy	Tagged Factor
gMgn	0.81	0.86	4.20	2.44	Gcn4
gMGn	0.85	NA	4.19	3.62	Gcn4
GmM	5.21	NA	NA	NA	Gcn4
GMMg	2.24	NA	NA	NA	Gcn4
GN	0.95	0.90	3.01	0.64	Gcn4
gNC	2.20	0.72	3.87	1.04	Gcn4
gNM	1.13	0.76	3.43	0.65	Gcn4
GNM	1.24	0.66	3.82	0.54	Gcn4
GnMm	3.92	NA	NA	1.21	Gcn4
Gnn	0.34	0.66	0.88	0.27	Gcn4
GNNc	1.68	0.79	2.71	0.78	Gcn4
m	1.22	0.92	1.51	0.16	Gcn4
M	1.49	0.95	1.68	0.17	Gcn4
mc	2.44	0.61	3.64	0.14	Gcn4
McG	2.64	1.28	NA	2.06	Gcn4
mCGC	2.89	1.82	NA	2.18	Gcn4
Mcggg	1.01	1.68	NA	12.71	Gcn4
mcgm	3.76	1.46	NA	2.51	Gcn4
mCm	4.78	0.45	NA	0.17	Gcn4
Mcm	4.29	NA	NA	NA	Gcn4
mCmC	3.68	0.56	4.37	0.15	Gcn4
Mcmgn	1.89	NA	NA	2.51	Gcn4
MCMMNc	4.09	1.15	NA	0.13	Gcn4

Promoter	Glucose Expression	Glucose Occupancy	AAS Expression	AAS Occupancy	Tagged Factor
MCN	2.09	0.63	2.77	0.13	Gcn4
McNcnm	2.88	NA	NA	NA	Gcn4
MCnn	1.83	0.76	1.81	0.16	Gcn4
mg	2.01	NA	NA	NA	Gcn4
mG	1.43	1.08	4.66	3.83	Gcn4
Mg	1.46	0.91	4.73	1.20	Gcn4
MG	1.71	1.06	5.28	1.07	Gcn4
mgc	2.59	1.55	NA	2.42	Gcn4
Mgc	2.95	1.37	NA	2.26	Gcn4
MGc	3.31	0.96	NA	2.22	Gcn4
mgCCnc	2.56	0.99	4.25	2.32	Gcn4
mgcmC	3.46	1.41	NA	3.55	Gcn4
MGg	1.06	1.03	NA	3.30	Gcn4
MggCc	1.78	NA	NA	NA	Gcn4
MGGcG	3.95	2.51	NA	19.41	Gcn4
MggCNM	1.95	NA	NA	NA	Gcn4
mggN	0.58	0.91	3.24	1.91	Gcn4
mgN	0.79	0.80	2.74	0.66	Gcn4
MGnGC	1.68	1.26	NA	5.93	Gcn4
mm	4.66	0.75	NA	0.18	Gcn4
MM	4.52	1.06	NA	0.16	Gcn4
mMccCG	2.82	NA	NA	2.78	Gcn4
MMGg	0.68	1.85	NA	10.92	Gcn4

Promoter	Glucose Expression	Glucose Occupancy	AAS Expression	AAS Occupancy	Tagged Factor
MMgmN	2.44	0.99	NA	1.40	Gcn4
mmn	2.80	0.62	5.00	0.12	Gcn4
MMNcg	1.18	NA	NA	NA	Gcn4
mmnmC	4.58	1.01	NA	0.20	Gcn4
Mn	0.67	0.72	1.04	0.14	Gcn4
MN	1.07	0.78	1.29	0.20	Gcn4
mNC	2.20	0.82	3.02	0.36	Gcn4
Mng	1.67	NA	NA	4.52	Gcn4
MNm	2.16	NA	3.44	NA	Gcn4
mNmC	3.17	0.64	NA	0.09	Gcn4
Mnmg	1.13	0.45	NA	0.65	Gcn4
MnMG	1.36	0.77	NA	1.08	Gcn4
MNMG	1.57	0.82	NA	1.01	Gcn4
mnMGG	0.75	NA	NA	4.43	Gcn4
MNnc	1.87	0.81	1.68	0.15	Gcn4
mnng	1.62	0.73	1.63	0.28	Gcn4
n	0.55	0.82	0.63	0.15	Gcn4
N	0.83	0.89	0.89	0.18	Gcn4
nc	2.02	0.67	1.79	0.14	Gcn4
nC	2.02	0.66	1.70	0.14	Gcn4
Nc	2.04	0.54	1.67	0.14	Gcn4
ncc	2.61	0.79	2.33	0.12	Gcn4
nCCGnm	1.84	1.41	NA	2.45	Gcn4

Promoter	Glucose Expression	Glucose Occupancy	AAS Expression	AAS Occupancy	Tagged Factor
ncg	1.33	NA	4.22	NA	Gcn4
NcG	2.30	1.26	NA	1.54	Gcn4
ncGgm	1.55	1.69	NA	4.72	Gcn4
nCGm	2.89	1.16	NA	1.78	Gcn4
NcGm	2.51	0.89	NA	1.60	Gcn4
ncGmm	5.22	1.58	NA	2.16	Gcn4
ncm	2.33	0.70	3.11	0.13	Gcn4
Ncm	2.61	0.67	4.48	0.23	Gcn4
ncmN	2.00	NA	NA	NA	Gcn4
nCmnGG	0.76	0.84	NA	6.15	Gcn4
ncN	1.58	NA	1.46	NA	Gcn4
nCnGm	1.25	1.03	NA	1.98	Gcn4
nCnm	1.85	0.79	3.00	0.14	Gcn4
nCNMCn	1.92	0.54	2.74	0.18	Gcn4
ng	0.56	0.86	2.48	1.08	Gcn4
Ng	0.71	1.34	3.82	1.30	Gcn4
NG	1.00	0.81	3.08	1.22	Gcn4
ngc	2.01	1.30	4.68	1.43	Gcn4
NGc	1.90	1.13	5.08	1.75	Gcn4
NGC	2.21	0.99	4.89	1.42	Gcn4
ngCMGC	2.70	1.49	NA	5.86	Gcn4
NGcNn	0.76	0.49	1.53	0.71	Gcn4
Ngg	0.76	0.91	3.46	2.46	Gcn4

Promoter	Glucose Expression	Glucose Occupancy	AAS Expression	AAS Occupancy	Tagged Factor
NGg	0.63	1.04	3.96	2.77	Gcn4
NggGc	1.27	1.93	NA	11.09	Gcn4
NgGMCm	2.69	2.99	NA	10.42	Gcn4
ngM	1.13	0.93	3.94	0.96	Gcn4
nGm	1.32	1.02	3.85	0.73	Gcn4
nGM	1.15	0.93	4.28	0.66	Gcn4
ngMc	2.35	1.13	NA	2.07	Gcn4
nGMC	3.00	NA	NA	7.94	Gcn4
NgMG	0.98	0.97	NA	3.52	Gcn4
ngMm	3.65	1.00	NA	1.28	Gcn4
ngMMn	1.92	0.60	NA	1.20	Gcn4
NgN	0.50	0.61	1.76	0.37	Gcn4
NgNGG	0.55	0.71	4.33	4.32	Gcn4
nm	0.87	0.97	1.16	0.32	Gcn4
Nm	1.20	0.83	1.37	0.22	Gcn4
NM	1.32	0.69	1.57	0.19	Gcn4
nMC	2.47	0.81	3.75	0.16	Gcn4
NMccNc	2.69	0.44	2.62	0.11	Gcn4
nmCNN	1.10	NA	2.08	NA	Gcn4
nmg	0.72	0.80	2.79	0.66	Gcn4
Nmg	1.03	1.18	3.51	1.33	Gcn4
nmGN	0.52	NA	2.12	NA	Gcn4
nmGng	0.99	0.64	3.86	1.99	Gcn4

Promoter	Glucose Expression	Glucose Occupancy	AAS Expression	AAS Occupancy	Tagged Factor
nMm	3.75	0.85	3.70	0.11	Gcn4
Nmn	2.74	NA	NA	NA	Gcn4
nmnM	1.79	0.61	3.26	0.19	Gcn4
nn	0.36	0.77	2.26	0.14	Gcn4
nN	0.62	NA	0.68	NA	Gcn4
Nn	0.52	NA	0.68	NA	Gcn4
NN	1.65	0.87	0.98	0.24	Gcn4
NNccc	2.73	0.52	2.47	0.09	Gcn4
NNCN	1.65	0.68	1.66	0.14	Gcn4
NNG	0.70	1.03	2.39	0.78	Gcn4
Nngc	1.63	1.41	4.85	1.35	Gcn4
nnGg	1.52	0.86	2.01	1.30	Gcn4
NNGn	0.34	0.50	1.37	0.27	Gcn4
Nnm	0.75	0.90	0.84	0.17	Gcn4
NnmG	0.63	0.59	3.10	0.98	Gcn4
nNN	0.65	NA	0.67	NA	Gcn4
NNn	0.35	NA	0.43	NA	Gcn4
cC	NA	0.71	NA	0.11	Gcn4
ccMmM	NA	0.83	NA	0.16	Gcn4
ccNgc	NA	2.11	NA	8.01	Gcn4
CMM	NA	0.65	NA	0.25	Gcn4
CNm	NA	0.97	NA	0.16	Gcn4
CNMM	NA	0.53	NA	0.09	Gcn4

Promoter	Glucose Expression	Glucose Occupancy	AAS Expression	AAS Occupancy	Tagged Factor
gCGGNN	NA	2.19	NA	7.08	Gcn4
gcgmGn	NA	1.49	NA	11.32	Gcn4
GGNmc	NA	1.07	NA	9.42	Gcn4
gMcNMM	NA	1.41	NA	3.48	Gcn4
GMGmn	NA	0.88	NA	2.89	Gcn4
gMM	NA	1.07	NA	2.64	Gcn4
gn	NA	1.56	NA	10.83	Gcn4
McNg	NA	1.08	NA	1.57	Gcn4
MGmM	NA	1.16	NA	2.46	Gcn4
MNgMmn	NA	1.06	NA	1.59	Gcn4
NCcmG	NA	1.99	NA	3.06	Gcn4
nG	NA	0.93	NA	1.35	Gcn4
NGCGNG	NA	1.52	NA	8.35	Gcn4
nGMGMC	NA	1.08	NA	6.40	Gcn4
nGMnCM	NA	0.68	NA	1.79	Gcn4
nGnm	NA	1.54	NA	6.41	Gcn4
NmmgMC	NA	1.88	NA	4.83	Gcn4
NNm	NA	0.75	0.45	0.13	Gcn4
Cn	NA	NA	NA	10.50	Gcn4
ggn	NA	NA	NA	15.99	Gcn4
GNgcCc	NA	NA	NA	8.89	Gcn4
gNGGNn	NA	NA	NA	3.54	Gcn4
mgmMNm	NA	NA	NA	8.88	Gcn4

Promoter	Glucose Expression	Glucose Occupancy	AAS Expression	AAS Occupancy	Tagged Factor
mmCN	NA	NA	NA	0.44	Gcn4
MmnNC	NA	NA	NA	0.09	Gcn4
MNcg	NA	NA	NA	7.37	Gcn4
mNm	NA	NA	NA	3.12	Gcn4
NcMM	NA	NA	NA	3.29	Gcn4
nMNMm	NA	NA	NA	2.66	Gcn4
NNmCNm	NA	NA	NA	0.08	Gcn4

Synthetic promoters were constructed and expression and occupancy values obtained as detailed in Methods. For promoters, C=Cbf1, fwd; c=Cbf1, rev; G=Gcn4, fwd; g=Gcn4, rev; M=Met31/Met32, fwd; m=Met31/Met32, rev; N=Nrg1, fwd; n=Nrg1, rev, where “fwd” and “rev” refer to the corresponding sequences in Table 3.6. Promoter sequences are listed from most distal to most proximal to the TSS of YFP. In the “Glucose Expression”, “Glucose Occupancy”, “AAS Expression”, and “AAS Occupancy” columns, NA means the value is not available. For Expression columns, this is due to the expression being out of the dynamic range of the cytometer. For Occupancy columns, this is due to their being too few reads in the IN sample to reliably estimate the input distribution.

Table S4: Overall fits and cross validation results

Data Used	# Parameters	Expression R^2	Occupancy R^2	Cross Validation
Expression-only	10	0.53	0.36	0.53
Occupancy-only	6	NA	0.57	0.57
Expression and Occupancy, noncompetitive	15	0.425	0.556	0.42 (expression)<br>0.56(occupancy)
Expression and Occupancy, competitive	15	0.431	0.554	0.43 (expression)<br>0.56 (occupancy)

Expression, occupancy, or expression and occupancy were modeled using the thermodynamic model described in Methods. Each model was fit with the number of parameters indicated (see Table 3.3 for specific parameter details). The model fit with only expression was also used to predict occupancy. The occupancy-only model cannot be used to predict expression since fitting of RNAP interaction terms was not attempted with only occupancy data. When fitting with expression and occupancy, the Gcn4 site was modeled without and with competitive binding (noncompetitive and competitive, respectively). One round of five-fold cross validation was performed on all models as described in the main text methods and the mean R^2 across the validations is reported in the Cross Validation column.

Table S5: Parameter values from thermodynamic model fitting

Fit Type	Parameter	Value (+/- 95% CI)
Expression only	$\Delta G_{Cbf1\text{-}DNA,glucose}$	1.32±0.71
Expression only	$\Delta G_{Met31/Met32\text{-}DNA,glucose}$	0.53±0.78
Expression only	$\Delta G_{Nrg1\text{-}DNA,glucose}$	0.41±0.47
Expression only	$\Delta G_{Cbf1,tagged-RNAP}$	-3.84±0.71
Expression only	$\Delta G_{Cbf1,untagged-RNAP}$	-1.14±0.72
Expression only	$\Delta G_{Gcn4,aas-RNAP}$	-1.55±0.32
Expression only	$\Delta G_{Gcn4,gluc-RNAP}$	0.48±0.33
Expression only	$\Delta G_{Met31/Met32-RNAP}$	-1.11±0.32
Expression only	$\Delta G_{Nrg1-RNAP}$	5.08±35.7
Expression only	$\Delta G_{RNAP\text{-}DNA}$	0.53±.28
Occupancy only	$\Delta G_{Cbf1\text{-}DNA,glucose}$	3.00±1.86
Occupancy only	$\Delta G_{Cbf1\text{-}DNA,AAS}$	2.91±1.87
Occupancy only	$\Delta G_{Gcn4\text{-}DNA,glucose}$	5.80± 2.17
Occupancy only	$\Delta G_{Gcn4\text{-}DNA,AAS}$	3.06± 1.82
Occupancy only	$\Delta G_{Gcn4\text{-}Gcn4}$	-2.62±1.24
Occupancy only	$\Delta G_{Gcn4\text{-}Nrg1}$	1.02±0.65
Expression and Occupancy	$\Delta G_{Cbf1\text{-}DNA,glucose}$	4.87*
Expression and Occupancy	$\Delta G_{Gcn4\text{-}DNA,glucose}$	2.92*
Expression and Occupancy	$\Delta G_{Met31/Met32\text{-}DNA,glucose}$	0.6*
Expression and Occupancy	$\Delta G_{Nrg1\text{-}DNA,glucose}$	-1.26*
Expression and Occupancy	$\Delta G_{Cbf1\text{-}DNA,AAS}$	4.95*
Expression and Occupancy	$\Delta G_{Gcn4\text{-}DNA,AAS}$	1.23*
Expression and Occupancy	$\Delta G_{RNAP\text{-}DNA}$	1.00*

Fit Type	Parameter	Value (+/- 95% CI)
Expression and Occupancy	$\Delta G_{Cbf1,tagged-RNAP}$	-5.36*
Expression and Occupancy	$\Delta G_{Gcn4-RNAP,glucose}$	17.65*
Expression and Occupancy	$\Delta G_{Met31/32-RNAP}$	-0.42*
Expression and Occupancy	$\Delta G_{Nrg1-RNAP}$	0.49*
Expression and Occupancy	$\Delta G_{Cbf1,untagged-RNAP}$	-3.15*
Expression and Occupancy	$\Delta G_{Gcn4-RNAP, AAS}$	-0.60*
Expression and Occupancy	$\Delta G_{Gcn4-Gcn4}$	-2.01*
Expression and Occupancy	$\Delta G_{Gcn4-Nrg1}$	3.00*
Expression and Occupancy, Competitive	$\Delta G_{Cbf1-DNA,Glucose}$	5.04±9.89
Expression and Occupancy, Competitive	$\Delta G_{Gcn4,Glucose}$	3.82±1.77
Expression and Occupancy, Competitive	$\Delta G_{Met31/Met32-DNA,glucose}$	0.41±1.30
Expression and Occupancy, Competitive	$\Delta G_{Nrg1-DNA,glucose}$	-0.93±1.49
Expression and Occupancy, Competitive	$\Delta G_{Cbf1-DNA,AAS}$	5.13±9.89
Expression and Occupancy, Competitive	$\Delta G_{Gcn4-DNA,AAS}$	0.64±0.34
Expression and Occupancy, Competitive	$\Delta G_{RNAP-DNA}$	0.92±0.22
Expression and Occupancy, Competitive	$\Delta G_{Cbf1,tagged-RNAP}$	-5.54±10.0
Expression and Occupancy, Competitive	$\Delta G_{Gcn4-RNAP,glucose}$	-0.82±0.34
Expression and Occupancy, Competitive	$\Delta G_{Met31/32-RNAP}$	-0.38±0.21
Expression and Occupancy, Competitive	$\Delta G_{Nrg1-RNAP}$	0.52±0.29
Expression and Occupancy, Competitive	$\Delta G_{Cbf1,untagged-RNAP}$	-3.3±9.63

Fit Type	Parameter	Value (+/- 95% CI)
Expression and Occupancy, Competitive	$\Delta G_{Gcn4\text{-}RNAP,\ AAS}$	0.31±0.22
Expression and Occupancy, Competitive	$\Delta G_{Gcn4\text{-}Gcn4}$	-1.78±0.56
Expression and Occupancy, Competitive	$\Delta G_{Gcn4\text{-}Nrg1}$	3.6±12.90

Confidence intervals were estimated using the “asympotic normal distribution for the parameter estimate“ method. For more details, see (65).

* Confidence interval estimates could not be calculated due to numerical instabilities introduced by the $\Delta G_{Gcn4\text{-}RNAP,Glucose}$ parameter.
